# On the crystal chemistry of inorganic nitrides: crystal-chemical parameters, bonding behavior, and opportunities in the exploration of their compositional space†

**DOI:** 10.1039/d0sc06028c

**Published:** 2021-02-15

**Authors:** Olivier C. Gagné

**Affiliations:** Earth and Planets Laboratory, Carnegie Institution for Science Washington D.C. 20015 USA ogagne@carnegiescience.edu

## Abstract

The scarcity of nitrogen in Earth's crust, combined with challenging synthesis, have made inorganic nitrides a relatively unexplored class of compounds compared to their naturally abundant oxide counterparts. To facilitate exploration of their compositional space *via a priori* modeling, and to help *a posteriori* structure verification not limited to inferring the oxidation state of redox-active cations, we derive a suite of bond-valence parameters and Lewis acid strength values for 76 cations observed bonding to N^3−^, and further outline a baseline statistical knowledge of bond lengths for these compounds. Examination of structural and electronic effects responsible for the functional properties and anomalous bonding behavior of inorganic nitrides shows that many mechanisms of bond-length variation ubiquitous to oxide and oxysalt compounds (*e.g.*, lone-pair stereoactivity, the Jahn–Teller and pseudo Jahn–Teller effects) are similarly pervasive in inorganic nitrides, and are occasionally observed to result in greater distortion magnitude than their oxide counterparts. We identify promising functional units for exploring uncharted chemical spaces of inorganic nitrides, *e.g.* multiple-bond metal centers with promise regarding the development of a post-Haber–Bosch process proceeding at milder reaction conditions, and promote an atomistic understanding of chemical bonding in nitrides relevant to such pursuits as the development of a model of ion substitution in solids, a problem of great relevance to semiconductor doping whose solution would fast-track the development of compound solar cells, battery materials, electronics, and more.

## Abstract 2

Navigating high-return chemical spaces in inorganic nitrides *via* identification of coordination units bearing functional properties.

## Graphical abstract

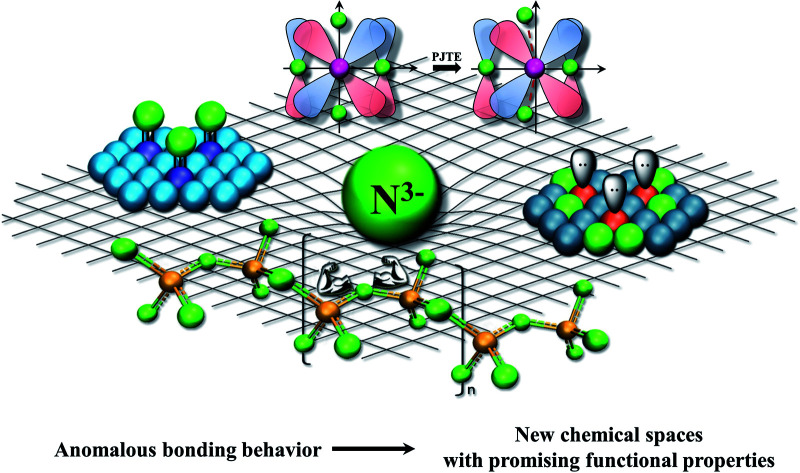

## Introduction

The predominance and variety of oxide and oxysalt compounds as minerals in Earth's crust means that they were inevitably among the first materials to be methodically characterized and examined for interesting properties, occupying scientists for decades in trying to decipher, reproduce, and enhance their behavior. Conversely, inorganic nitrides, here defined as ionic/covalent compounds with N^3−^ as the main anion, are a relatively unexplored class of compounds owing to (1) the scarcity of nitrogen minerals in Earth's crust due to various biological and geological transport processes drawing nitrogen away from the crust^1^ (not limited to their reaction with water to form hydroxides and ammonia^2^), and (2) challenging synthesis.^3^

Systematic investigation of nitrides began in the late 1930s with the work of Juza and collaborators who mainly focused on lithium compounds for their relative ease of preparation.^4–6^ Rapid progression in the synthesis and characterization of nitride compounds followed the introduction of several new methods of preparation in the 1980s. This sudden burst of interest eventually led to the successful synthesis of many compounds suited to single-crystal X-ray diffraction, resulting in collection of significantly more accurate bond lengths compared to those determined *via* powder diffraction.^7^ Several reviews on the descriptive crystal chemistry of inorganic nitrides followed^2,8–14^ (more recent reviews include those of Höhn & Niewa for non-main group elements,^15^ and Tareen *et al.* for mixed ternary transition metal nitrides^16^), and the chemistry of nitrides was soon likened to that of silicates;^10^ the term *nitridometalate* was introduced to describe compounds containing covalent complex anions [M_*x*_N_*y*_]^*z*−^ (in relation to oxometallates, commonly called oxyanions in the Earth sciences),^11,12^ reflecting the richness of chemistries to come. New classes of inorganic nitrides that were initially considered to be scientific curiosities have since been described as functionally diverse groups of materials, not limited to the nitridosilicates,^8,17^ oxynitrididosilicates^18^ and perovskite-related oxynitrides.^19,20^

In parallel to developments occurring in the bulk, the past decades saw binary III–V nitrides gain notoriety as semiconductors due to a trove of desirable properties largely associated with their wide, direct, and highly tunable band gaps. These properties include high temperature and power operation, high breakdown voltages, high thermal conductivity, high phonon frequency, low noise generation, and resilience to both large electric fields and hostile thermal/chemical environments, thus making these compounds attractive for a wide range of electronic and optoelectronic applications of commercial and industrial interest.^21–26^ More recently, ternary II–IV-N_2_ nitrides were introduced,^27–29^ thus providing additional compositional (and structural) flexibility for fine-tuning these properties.

Nitrides *sensu lato* provide a great opportunity for materials discovery owing to their unique electronic and bonding characteristics. Large-scale computational and synthetic efforts are underway to explore their compositional space. These compounds are being investigated for properties arising from both their bulk and trace/minor element composition spanning energy conversion and storage,^30^ solar-driven CO_2_ reduction (*e.g.* in Z-scheme-inspired photoelectrochemical cells),^31^ in quantum information processing,^32^ as alternatives to metals, metal oxides and metal sulfides in heterogeneous catalysis,^33^ as piezoelectric^34,35^ and photoluminescent^36^ materials, electrocatalysts,^37,38^ electrochemical sensors,^39^ photocatalysts,^40,41^ photovoltaics,^42–44^ photodetectors,^45,46^ light-emitting diodes,^47,48^ thermoelectrics,^34,49^ superconductors,^50–52^ as hard coating^53^ and ultrahard materials^54^ (in their pernitride form), *etc.*, demonstrating the importance of an adequate understanding of their chemical bonding – a feat typically achieved in the bulk, and applied locally in studying point defects. With a relatively slow start compared to oxides and oxysalts, it is no surprise that some of the most exciting properties of these materials are currently being realized, and that much promise lies ahead in the exploratory synthesis of functional inorganic nitrides.

The present work is premised on a distillation of crystallographic knowledge for inorganic nitrides, with the goal of facilitating their characterization and design. Such pursuits have been eased by the bond-valence model for many classes of inorganic compounds, which is for example used to screen compounds in pymatgen^55^ and to infer the oxidation state of redox-active ions^56^ under the umbrella of the Materials Project.^57^ Most recently, the bond-topological nature of the bond-valence model was featured in showing how bond-length variation (thus polyhedral distortion) is an inherent, predictable and quantifiable by-product of chemical bonding in inorganic solids^58^ (polyhedral distortion having crucial implications with regard to the functional properties of various types of materials not limited to ferroelectricity,^59,60^ piezoelectricity,^59,61^ flexoelectricity,^62^ second-order nonlinear optical behavior,^59,63^ negative thermal expansion,^64^ and photoluminescence^65,66^). However, the parameterization of the bond-valence model is largely incomplete for cations bonded to N^3−^, and the quality of published bond-valence parameters is not established. In this work, we use the method of Gagné & Hawthorne^67^ to derive new bond-valence parameters for nitrides, provide a scale of Lewis acidity for nitrides, and further outline a baseline statistical knowledge of bond lengths for cations bonded to N^3−^; these data serve as a contribution to our gradual efforts of systematizing chemical bonding behavior in solids, toward modernizing Shannon's set of ionic radii^68^ (see ref. 58 and 69–72 for bond-length statistics of cations bonded to O^2−^; *in prep* for cations bonded to S^2−^, Se^2−^ and/or Te^2−^), and are a useful aid to structure verification and the design of physically realistic crystal structures. In the second part of this work, we explore new venues for the exploratory synthesis of functional inorganic nitrides, using the systematic nature of this dataset to identify plausible ion configurations likely to lead to new and promising functional units to be transposed across various chemical spaces. Considering that the pairwise bonding data given herein are independent of their chemical environment, the type of analysis advanced in this work may be extended to other fields whose functional units may differ from those covered in this article, *e.g.* coordination chemistry, mineralogy, biochemistry, *etc.*

## Dataset

We used the Inorganic Crystal Structure Database (ICSD) to extract bond-length data for elements bonded to N^3−^ as a function of oxidation state and coordination number. Data collection criteria are those outlined by Gagné & Hawthorne:^69^ (1) publication date ≥ 1975; (2) *R*_1_ ≤ 0.06; (3) the site of interest is fully occupied by the cation; (4) all bonds involve ions at fully occupied sites; (5) the cation and anion sites of interest show no positional disorder; (6) crystallographic data were measured at ambient conditions; (7) no data from powder, electron or synchrotron diffraction were included; (8) for H, only neutron-diffraction data were collected. The procedure used to determine the coordination polyhedron in ambiguous cases is also that of Gagné & Hawthorne.^69^

Following data collection, we examined structures with questionable bond-lengths and/or mean bond-lengths for various problems (*e.g.* positional/substitutional disorder, inconsistent (an)isotropic displacement parameters, high standard deviations on bond lengths, *etc.*) and discarded data which could not be confidently confirmed.

Whereby bond-valence parameters may be derived for mixed-anion coordination polyhedra (see ESI†), we also collected bonding data for cations bonded to both N^3−^ and O^2−^; the final dataset used to derive the bond-valence parameters given in Table 1 consists of 6770 bond lengths hand-picked from 1436 coordination polyhedra from 720 crystal-structure refinements, covering 76 cations bonded to N^3−^ (and possibly also O^2−^). The dataset that omits mixed-anion data accounts for 4048 bond lengths taken from 875 cation coordination polyhedra; these data and their basic statistics are reported in Table 2 and will be discussed further below.

**Table 1:** Lewis acid strengths (*S*_a_) and bond-valence parameters (*R*_o_, *B*) for cations bonded to N^3−^

Ion	No. of coordination polyhedra	Average observed coordination number^b^	*S*_a_ (v.u.)	Std. dev. on *S*_a_	*R*_o_ (Å)	*B* (Å)	RMSD (v.u.)		Method of derivation (1-CN ions)^c^
aH^+^	30				0.935	0.572	0.032		
Li^+^	83	3.63 (6)	0.275 (4)	0.070	1.713	0.312	0.111		
Be^2+^	2	3.5 (2)	0.57 (3)	0.08	1.537	0.301	0		
B^3+^	51	2.43 (6)	1.24 (3)	0.33	1.467	0.321	0.079		
C^4+^	270	2.76 (2)	1.45 (1)	0.22	1.401	0.261	0.094		
N^5+^	6	2	2.5		1.51	0.345	0.027		
Na^+^	74	5.29 (6)	0.189 (2)	0.027	1.62	0.546	0.098		
Mg^2+^	7	4	0.5		1.83	0.37	0.252		
Al^3+^	11	4.20 (9)	0.71 (2)	0.10	1.772	0.413	0.187		
Si^4+^	58	4.02 (1)	0.995 (2)	0.036	1.742	0.422	0.175		3
P^5+^	80	4	1.25		1.72	0.414	0.307		
S^4+^	10	2	2		1.781	0.328	0.081		2
S^6+^	46				1.731	0.366	0.075		
K^+^	67	6.46 (9)	0.155 (2)	0.028	1.892	0.543	0.122		
Ca^2+^	43	5.13 (7)	0.390 (5)	0.074	2.114	0.435	0.214		
V^2+^	1	6	1/3		1.779	0.405	—		1
V^3+^	2	4.5 (5)	0.67 (7)	0.22	1.815	0.33	0		
V^5+^	1	4	1.25		1.93	0.399	—		2
Cr^2+^	1	6	1/3		1.816	0.372	—		1
Cr^3+^	26	5.8 (1)	0.522 (9)	0.075	1.796	0.403	0.061		
Cr^5+^	1	4	1.25		1.844	0.399	—		2
Cr^6+^	2	4	1.5		1.924	0.399	0.075		2
Mn^2+^	25	5.3 (1)	0.377 (9)	0.064	1.874	0.328	0.141		
Mn^3+^	3	3	1		1.759	0.399	0.124		2
Mn^5+^	2	4	1.25		1.906	0.399	0.063		2
Fe^2+^	15	5.2 (2)	0.39 (1)	0.10	1.719	0.427	0.084		
Fe^3+^	3	4.3 (3)	0.69 (6)	0.20	1.74	0.687	0.059		
Co^+^	1	2	0.5		1.472	0.399	—		2
Co^2+^	9	4.7 (2)	0.429 (1)	0.09	1.626	0.485	0.049		
Co^3+^	66	6	0.5		1.686	0.399	0.062		2
Ni^2+^	19	4.6 (1)	0.44 (1)	0.09	1.611	0.457	0.054		
Cu^+^	9	2.2 (1)	0.45 (3)	0.13	1.539	0.399	0.2		2
Cu^2+^	17	5.5 (2)	0.36 (1)	0.06	1.577	0.515	0.129		
Zn^2+^	29	3.85 (7)	0.51 (1)	0.10	1.792	0.293	0.264		
Ga^3+^	4	4.5 (2)	0.67 (3)	0.13	1.858	0.318	0.164		
Ge^4+^	2				1.891	0.422	0.014		3
Se^6+^	2				1.945	0.422	0.067		3
Rb^+^	29	7.7 (1)	0.130 (2)	0.023	1.914	0.639	0.039		
Sr^2+^	32	5.8 (2)	0.35 (1)	0.12	2.269	0.441	0.215		
Y^3+^	5	6	0.5		2.114	0.399	0.055		2
Nb^5+^	12	4.3 (1)	1.15 (3)	0.20	2.052	0.404	0.057		
Mo^6+^	13	4	1.5		1.97	0.265	0.129		
Ru^3+^	8	6	0.5		1.816	0.399	0.02		2
Rh^3+^	5	6	0.5		1.795	0.399	0.023		2
Pd^2+^	5	4	0.5		1.767	0.399	0.033		2
Ag^+^	18	2.5 (1)	0.41 (2)	0.12	1.926	0.277	0.089		
Cd^2+^	8	6	1/3		1.889	0.399	0.048		2
Sn^2+^	1	3	2/3		1.965	0.438	—		2
Sn^4+^	4	6	2/3		2	0.438	0.003		2
Cs^+^	24	9.5 (1)	0.106 (1)	0.021	1.979	0.67	0.077		
Ba^2+^	53	6.68 (9)	0.299 (4)	0.067	2.432	0.405	0.171		
La^3+^	21	8.00 (0.08)	0.375 (4)	0.037	2.177	0.52	0.192		
Ce^3+^	11	8.40 (8)	0.357 (3)	0.021	2.162	0.469	0.157		2
Ce^4+^	1	6	2/3		2.237	0.469	—		2
Pr^3+^	3	7	3/7		2.129	0.469	0.183		2
Nd^3+^	8	6.7 (2)	0.45 (1)	0.06	2.051	0.555	0.256		
Sm^3+^	7	7	3/7		2.042	0.469	0.119		2
Eu^2+^	5	8.4 (4)	0.24 (1)	0.07	1.952	0.587	0.089		
Eu^3+^	3	6	0.5		2.238	0.275	0.279		
Gd^3+^	2				2.064	0.38	0.017		
Tb^3+^	3				2.042	0.415	0.019		
Dy^3+^	1				1.978	0.469	—		2
Ho^3+^	2	6	0.5		2.097	0.391	0.25		1
Er^3+^	4	6	0.5		2.057	0.498	0.138		
Yb^3+^	3	6.3 (1)	0.474 (8)	0.035	1.928	0.584	0.19		
Lu^3+^	1	6	0.5		1.966	0.487	—		1
Hf^4+^	1	8	0.5		2.023	0.399	—		2
Ta^5+^	5	4	1.25		2.047	0.399	0.038		2
W^6+^	27	4	1.5		2.026	0.399	0.089		2
Pt^2+^	15	4	0.5		1.817	0.351	0.063		
Au^+^	2	2	0.5		1.743	0.399	0.187		2
Tl^+^	8	6.88 (8)	0.145 (2)	0.013	2.114	0.493	0.03		
Pb^2+^	2	6.0 (3)	0.33 (2)	0.06	2.058	0.529	0		
Bi^3+^	3				2.066	0.438	0.018		2
U^4+^	1	8	0.5		2.129	0.422	—		3
U^6+^	2				2.035	0.422	0.053		3
Mean RMSD *n* ≥ 10								0.122	
Mean RMSD weighted by number of CP								0.120	

a Neutron-diffraction data.

b Data from mixed-anion coordination polyhedra are omitted in the calculation of AOCN and *S*_a_; more detail on these data are given in Table 2.

c 1: *R*_o_ fixed to predicted value, 2: *B* fixed to family average, 3: *B* fixed to 0.399 Å.

**Table 2:** Bond-length statistics for cations bonded to N^3−^

Ion	Coordination number	Number of coordination polyhedra^a^	Number of bonds	Mean bond-length (Å)	Standard deviation (Å)	Range (Å)	Maximum bond-length (Å)	Minimum bond-length (Å)
H^+^	2	0 (29)	0 (58)					
	3	0 (1)	0 (3)					
Li^+^	2	11	22	1.947	0.025	0.078	1.990	1.912
	3	12	36	2.121	0.029	0.125	2.181	2.056
	4	43 (10)	172 (40)	2.148	0.103	0.690	2.613	1.923
	5	2 (2)	10 (10)	2.240	0.185	0.681	2.722	2.041
	6	3	18	2.255	0.039	0.094	2.302	2.208
Be^2+^	3	1	3	1.660	0.026	0.059	1.683	1.624
	4	1	4	1.746	0.014	0.038	1.770	1.732
B^3+^	2	31	62	1.337	0.017	0.096	1.383	1.287
	3	12 (3)	36 (9)	1.473	0.029	0.143	1.545	1.402
	4	4 (1)	16 (4)	1.557	0.019	0.052	1.570	1.518
C^4+^	2	33 (4)	66 (8)	1.226	0.061	0.188	1.325	1.137
	3	103 (130)	309 (390)	1.326	0.017	0.119	1.395	1.276
N^5+^	2	2	4	1.194	0.000	0.000	1.194	1.194
	3	0 (4)	0 (12)					
Na^+^	4	6 (5)	24 (20)	2.417	0.052	0.200	2.538	2.338
	5	12 (7)	60 (35)	2.594	0.196	0.783	3.116	2.333
	6	16 (26)	96 (156)	2.562	0.113	0.640	3.022	2.382
	7	0 (2)	0 (14)					
Mg^2+^	4	4	16	2.121	0.049	0.162	2.198	2.036
	6	0 (3)	0 (18)					
Al^3+^	4	9	36	1.898	0.043	0.144	1.984	1.840
	6	1 (1)	6 (6)	2.044	0.006	0.015	2.052	2.037
Si^4+^	4	46 (11)	184 (44)	1.738	0.039	0.288	1.941	1.653
	5	1	5	1.943	0.259	0.588	2.287	1.699
P^5+^	4	28 (52)	112 (208)	1.624	0.030	0.151	1.711	1.560
S^4+^	2	9 (1)	18 (2)	1.552	0.020	0.069	1.596	1.527
S^6+^	4	0 (46)	0 (184)					
K^+^	4	1	4	2.688	0.025	0.061	2.709	2.648
	5	1	5	2.907	0.165	0.424	3.134	2.710
	6	19 (6)	114 (36)	2.894	0.112	0.509	3.210	2.701
	7	0 (12)	0 (84)					
	8	6 (15)	48 (120)	3.115	0.236	0.898	3.703	2.805
	9	0 (6)	0 (54)					
	10	1	10	3.083	0.305	0.844	3.638	2.794
Ca^2+^	4	9	36	2.491	0.031	0.115	2.526	2.411
	5	16 (1)	80 (5)	2.526	0.113	0.510	2.823	2.313
	6	11 (4)	66 (24)	2.563	0.107	0.616	2.965	2.349
	7	0 (1)	0 (7)					
	9	1	9	2.770	0.072	0.155	2.823	2.668
V^2+^	6	1	6	2.224	0.000	0.000	2.224	2.224
V^3+^	3	1	3	1.815	0.007	0.017	1.825	1.808
	6	1	6	2.044	0.005	0.012	2.051	2.039
V^5+^	4	1	4	1.842	0.016	0.038	1.851	1.813
Cr^2+^	6	1	6	2.224	0.000	0.000	2.224	2.224
Cr^3+^	3	1	3	1.798	0.046	0.097	1.863	1.766
	6	11 (14)	66 (84)	2.078	0.025	0.193	2.158	1.965
Cr^5+^	4	1	4	1.755	0.002	0.004	1.757	1.753
Cr^6+^	4	2	8	1.763	0.013	0.031	1.780	1.749
Mn^2+^	4	3	12	2.109	0.032	0.095	2.166	2.071
	5	1 (1)	5 (5)	2.306	0.326	0.827	2.958	2.131
	6	6 (14)	36 (84)	2.241	0.044	0.247	2.417	2.170
Mn^3+^	3	3	9	1.760	0.025	0.059	1.798	1.739
Mn^5+^	4	2	8	1.818	0.007	0.015	1.825	1.810
Fe^2+^	3	3	9	1.899	0.026	0.080	1.941	1.861
	4	1	4	2.008	0.000	0.000	2.008	2.008
	6	9 (2)	54 (12)	2.196	0.032	0.128	2.274	2.146
Fe^3+^	3	1	3	1.730	0.000	0.000	1.730	1.730
	4	1	4	1.957	0.000	0.000	1.957	1.957
	6	1	6	2.207	0.000	0.000	2.207	2.207
Co^+^	2	1	2	1.749	0.000	0.000	1.749	1.749
Co^2+^	4	4	16	1.963	0.011	0.035	1.983	1.948
	6	2 (3)	12 (18)	2.181	0.005	0.010	2.186	2.176
Co^3+^	6	36 (30)	216 (180)	1.963	0.013	0.115	2.007	1.892
Ni^2+^	4	10	40	1.928	0.025	0.141	2.014	1.873
	6	4 (5)	24 (30)	2.127	0.026	0.085	2.164	2.079
Cu^+^	2	8	16	1.875	0.022	0.084	1.931	1.847
	4	1	4	1.977	0.000	0.000	1.977	1.977
Cu^2+^	4	1 (1)	4 (4)	1.941	0.012	0.032	1.953	1.921
	5	0 (2)	0 (10)					
	6	3 (10)	18 (60)	2.185	0.288	0.843	2.722	1.879
Zn^2+^	2	3	6	1.860	0.013	0.032	1.874	1.842
	4	23 (2)	92 (8)	1.984	0.038	0.180	2.086	1.906
	6	1	6	2.136	0.000	0.000	2.136	2.136
Ga^3+^	4	3	12	1.952	0.040	0.159	2.063	1.904
	6	1	6	2.079	0.012	0.035	2.099	2.064
Ge^4+^	4	0 (2)	0 (8)					
Se^6+^	4	0 (2)	0 (8)					
Rb^+^	6	6	36	3.080	0.104	0.441	3.347	2.906
	7	1 (1)	7 (7)	3.085	0.099	0.315	3.191	2.876
	8	14 (3)	112 (24)	3.263	0.193	0.806	3.764	2.958
	9	0 (1)	0 (9)					
	10	1	10	3.484	0.308	0.804	3.915	3.111
	11	0 (1)	0 (11)					
	12	1	12	3.568	0.283	0.605	3.773	3.168
Sr^2+^	4	9	36	2.641	0.030	0.137	2.682	2.545
	5	6	30	2.691	0.122	0.460	2.940	2.480
	6	9 (1)	54 (6)	2.749	0.150	0.708	3.216	2.508
	7	1 (1)	7 (7)	2.916	0.229	0.601	3.221	2.620
	8	1 (1)	8 (8)	2.977	0.299	0.811	3.379	2.568
	10	2	20	2.999	0.248	0.810	3.380	2.570
	13	1	13	3.268	0.311	1.024	3.730	2.706
Y^3+^	6	5	30	2.394	0.050	0.174	2.478	2.304
Nb^5+^	4	10	40	1.963	0.024	0.110	2.026	1.916
	6	2	12	2.126	0.003	0.006	2.129	2.123
Mo^6+^	4	11 (1)	44 (4)	1.864	0.031	0.149	1.951	1.802
	5	0 (1)	0 (5)					
Ru^3+^	6	8	48	2.092	0.005	0.019	2.101	2.082
Rh^3+^	6	3 (2)	18 (12)	2.071	0.007	0.024	2.078	2.054
Pd^2+^	4	5	20	2.044	0.015	0.060	2.079	2.019
Ag^+^	2	9 (2)	18 (4)	2.125	0.026	0.108	2.192	2.084
	3	2	6	2.266	0.099	0.295	2.445	2.150
	4	2	8	2.277	0.024	0.085	2.316	2.231
	5	0 (1)	0 (5)					
	6	0 (2)	0 (12)					
Cd^2+^	6	4 (4)	24 (24)	2.341	0.044	0.227	2.466	2.239
Sn^2+^	3	1	3	2.143	0.019	0.042	2.170	2.128
Sn^4+^	6	4	24	2.177	0.000	0.001	2.178	2.177
Cs^+^	6	1	6	3.187	0.000	0.000	3.187	3.187
	7	2	14	3.342	0.135	0.393	3.549	3.156
	8	5 (1)	40 (8)	3.396	0.146	0.641	3.818	3.177
	9	2 (1)	18 (9)	3.479	0.242	0.839	3.940	3.101
	10	5	50	3.559	0.185	0.836	4.034	3.198
	11	1	11	3.665	0.242	0.698	4.068	3.370
	12	5 (1)	60 (12)	3.642	0.145	0.448	3.827	3.379
Ba^2+^	4	1	4	2.738	0.065	0.155	2.798	2.643
	5	6 (1)	30 (5)	2.868	0.088	0.388	3.041	2.653
	6	13 (1)	78 (6)	2.896	0.156	0.768	3.375	2.607
	7	16	112	2.960	0.177	0.771	3.447	2.676
	8	7	56	3.059	0.234	0.846	3.519	2.673
	9	0 (3)	0 (27)					
	10	0 (3)	0 (30)					
	11	0 (1)	0 (11)					
	14	1	14	3.293	0.226	0.665	3.490	2.825
La^3+^	6	1 (1)	6 (6)	2.531	0.006	0.013	2.535	2.522
	7	1 (1)	7 (7)	2.607	0.151	0.397	2.834	2.437
	8	8 (3)	64 (24)	2.698	0.104	0.553	2.965	2.412
	9	3 (3)	27 (27)	2.770	0.154	0.621	3.098	2.477
Ce^3+^	8	3 (2)	24 (16)	2.646	0.120	0.486	2.849	2.363
	9	2 (4)	18 (36)	2.740	0.192	0.713	3.133	2.420
Ce^4+^	6	1	6	2.427	0.000	0.000	2.427	2.427
Pr^3+^	7	1	7	2.572	0.081	0.236	2.691	2.455
	8	0 (1)	0 (8)					
	9	0 (1)	0 (9)					
Nd^3+^	6	2	12	2.497	0.062	0.164	2.571	2.407
	8	1 (3)	8 (24)	2.589	0.005	0.010	2.594	2.584
	9	0 (2)	0 (18)					
Sm^3+^	7	1	7	2.477	0.046	0.162	2.544	2.382
	8	0 (5)	0 (40)					
	9	0 (1)	0 (9)					
Eu^2+^	6	2	12	2.599	0.106	0.279	2.715	2.436
	8	1	8	2.861	0.256	0.681	3.235	2.554
	10	1	10	2.928	0.214	0.717	3.246	2.529
	12	1	12	2.993	0.048	0.135	3.059	2.924
Eu^3+^	6	1	6	2.463	0.002	0.003	2.464	2.461
	8	0 (1)	0 (8)					
	9	0 (1)	0 (9)					
Gd^3+^	9	0 (2)	0 (18)					
Tb^3+^	8	0 (3)	0 (24)					
Dy^3+^	8	0 (1)	0 (8)					
Ho^3+^	6	1	6	2.417	0.104	0.274	2.609	2.335
	8	0 (1)	0 (8)					
Er^3+^	6	1 (2)	6 (12)	2.401	0.056	0.165	2.525	2.360
	8	0 (2)	0 (16)					
Yb^3+^	6	2	12	2.366	0.046	0.124	2.417	2.293
	7	1	7	2.397	0.138	0.403	2.664	2.261
Lu^3+^	6	1	6	2.304	0.021	0.042	2.325	2.283
Hf^4+^	8	1	8	2.327	0.148	0.295	2.474	2.179
Ta^5+^	4	5	20	1.959	0.027	0.106	2.008	1.902
W^6+^	4	26 (1)	104 (4)	1.868	0.054	0.218	1.972	1.754
Pt^2+^	4	4 (11)	16 (44)	2.049	0.008	0.030	2.065	2.035
Au^+^	2	2	4	2.020	0.006	0.013	2.025	2.012
Tl^+^	6	2	12	3.039	0.167	0.571	3.385	2.814
	7	5	35	3.117	0.240	0.913	3.664	2.751
	8	1	8	3.204	0.239	0.762	3.542	2.780
Pb^2+^	5	1	5	2.559	0.125	0.313	2.622	2.309
	7	1	7	2.724	0.064	0.155	2.822	2.667
Bi^3+^	8	0 (3)	0 (24)					
U^4+^	8	1	8	2.424	0.040	0.080	2.464	2.384
U^6+^	7	0 (2)	0 (14)					

a Numbers in parentheses are for mixed (O^2−^ and N^3−^) coordination polyhedra. Statistical results given here do not include these data.

## Derivation of bond-valence parameters

While the first proposal of a relation between bond length and bond strength can be attributed to Pauling,^73^ the first *universal* two-body correlation between these variables was described by Brown and Shannon^74^ in what ultimately developed into the bond-valence model.^75^ The bond-valence relation was initially proposed as an inverse power equation^74^ and later reformulated to1
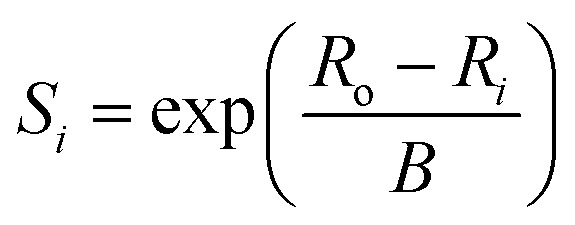
where *R*_*i*_ is the bond length, *S*_*i*_ is the bond valence, and *R*_o_ and *B* are bond-valence parameters, derived on the basis of ion pair.^76^ A principal axiom of the bond-valence model, which notably serves as a basis for deriving bond-valence parameters, is the valence-sum rule. The valence-sum rule states that *the sum of the bond valences at each atom is equal to the magnitude of the atomic valence*,^75^2
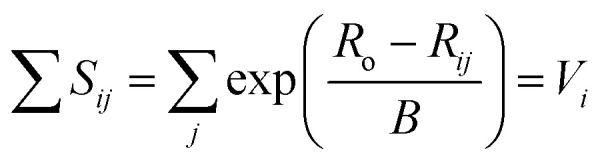
where the sum is taken over the *j* nearest neighbours of cation *i*, and where *V*_*i*_ is the atomic valence (oxidation state) of cation *i*. Following a review of methods used for the derivation of bond-valence parameters, Gagné & Hawthorne proposed the *Generalized Reduced Gradient (GRG) method of RMSD minimization* for the derivation of new bond-valence parameters^67^ (where the RMSD is from the valence-sum rule, in valence units, v.u.). This method uses the GRG algorithm to find the global minimum of3
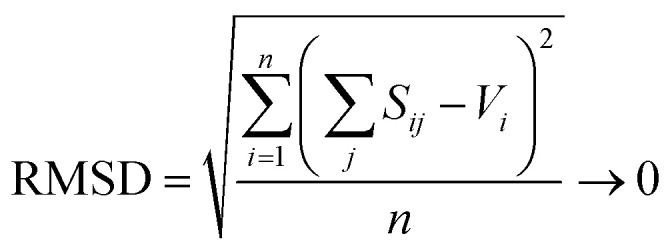
where the minimization is done over *n* observed coordination polyhedra for a given cation–anion pair. Gagné & Hawthorne further propose the use of a weighting scheme that finds a balance between overall fit (RMSD; eqn (3)) and fit on the basis of cation coordination number4
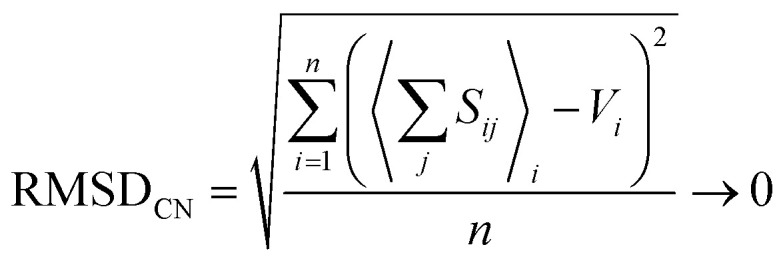
where the term between brackets is the mean bond-valence sum on the basis of coordination number, and where the summation is done over *n* observed coordination numbers. This added term ensures that a potentially disproportionate amount of data for a given cation coordination number does not overtake the optimization procedure, which can otherwise result in faulty bond-valence parameters. We used the 2 : 1 weighting scheme between RMSD and RMSD_CN_ of Gagné & Hawthorne to derive the bond-valence parameters of Table 1. A detailed discussion of the method used to derive bond-valence parameters for mixed-anion cation coordination polyhedra, and those observed in a single coordination number, is given in the ESI† alongside complementary verification of anion bond-valence sums for the full set of parameters.

### Comparison to O^2−^ bond-valence parameters

For the 40 cations for which both *R*_o_ and *B* were refined, the mean increase in *R*_o_ and *B* are 0.064 and 0.023 Å, respectively, in comparison to the parameters of Gagné & Hawthorne.^67^ When weighting these changes by the number of coordination polyhedra used for each ion, these numbers are 0.092 and −0.011 Å, respectively. The largest change is for Li^+^: for O^2−^, *R*_o_ = 1.062, *B* = 0.642 Å, and for N^3−^, *R*_o_ = 1.713, *B* = 0.312 Å. This variation is an artifact of a shallow RMSD global minimum (typical of alkali and alkaline-earth metals), whereby large changes in the bond-valence parameters lead to only slight changes in RMSD. A lower value of *R*_o_ for N^3−^*vs.* O^2−^ is usually associated with an increased value for *B*, and *vice versa*.

### Comparison with published bond-valence parameters

We compared the parameters of this work to the set of *soft* bond-valence parameters of Chen & Adams^77^ which they adapted to the first coordination shell (but did not evaluate for anion bond-valence sums). Fewer bond-valence parameters are given by these authors for nitrides, allowing comparison for 25 pairs of bond-valence parameters. Whereby the valence-sum rule applies equally to cations and anions, and good agreement for cation bond-valence sums (BVS) in no way implies good agreement for anion BVS, our evaluation necessarily covers both cation and anion BVS. For a set of 25 reliable crystal structures selected for this purpose, the mean anion RMSD observed for the parameters of this work and those of Chen & Adams are 0.236 v.u. and 0.258 v.u., respectively. For cation BVS, the mean cation RMSD over the 25 ion pairs is 0.118 v.u. for the parameters of this work, and 0.219 v.u. for the parameters of Chen & Adams. Weighting by the number of coordination polyhedra, these values are 0.136 and 0.369 v.u., respectively, thus validating the derivation method of Gagné & Hawthorne.^67^

### On deriving bond-valence parameters

We stress that there is no good alternative to ensuring the quality of bond-valence parameters other than to verify their performance over a large number of crystal-structure refinements.

With strong correlation between the ratio of bond-valence parameter *R*_o_ and mean-bond-length as a function of the *n*^th^ ionization energy of the cation,^67^ it may be tempting to derive bond-valence parameters for ion pairs without empirical data taking the mean-bond-length to be equal to the sum of the constituent ionic radii. However, this practice is fraught with uncertainty due to (1) large uncertainty associated with Shannon's ionic radii (the subject of upcoming work), and (2) the risk involved in fixing bond-valence parameter *R*_o_ instead of *B* (even where experimental data are available).^67^ It is for these reasons that we have refrained from predicting bond-valence parameters which cannot be verified *via* high-quality experimental data. Even where such data are available, one may easily be misled into reporting “high-quality” bond-valence parameters if their quality is not checked against anion bond-valence sums.

## Derivation of Lewis acid strengths

Pearson's concept of hard and soft acids and bases (HSAB)^78,79^ may be conveniently transposed and quantified onto the bond-valence scale as

5 *S*_a_ = *V*/*N*_*i*_

where *S*_a_ is the Lewis acid strength of a cation (analogously, *S*_b_ is the Lewis base strength of anions), *V* is the oxidation state, and *N*_i_ is the average observed coordination number (AOCN) of the cation compiled over a large number of crystal structures.^80^ As such, the Lewis acidity of a cation may be interpreted as the mean observed bond-valence of a cation (or cation group) when bonded to a specific anion (or anion group). Furthermore, its standard deviation (calculated from that of the AOCN) may be interpreted as the ability of the cation to adjust to a range of Lewis base strengths *via* the valence-matching principle. The valence-matching principle states that the most stable structures will form when the Lewis acid strength of the cation closely matches the Lewis base strength of the anion (with island of stability 0.5 < *S*_a_/*S*_b_ < 2).^75,81^ This argument has notably been used to predict the weakly bonded constituents (interstitial complex) that link strongly bonded oxyanions to form the crystal structures of minerals,^82^ and to explain the distribution of mineral stoichiometries in nature.^83,84^ Foreseeably, the concept of Lewis acidity/basicity may also be used in modeling, *e.g.* in crystal-structure prediction and materials design.

As Lewis acid strength is dependent upon the base against which it is measured,^85^ we derive a scale of Lewis acidity for 77 cations bonded to N^3−^ (Table 1) to complement the dataset of Gagné & Hawthorne for cations bonded to O^2−^.^86^ On average, Lewis acid strength values increase by 0.115 v.u. for cations bonded to N^3−^*vs.* O^2−^, due to generally lower observed cation coordination numbers. Notable discrepancies result from a favored tetrahedral over octahedral coordination for such ions as Nb^5+^, Mo^6+^, and W^6+^ when bonded to N^3−^.

### Correlation with ionization energy

**Fig. 1:**
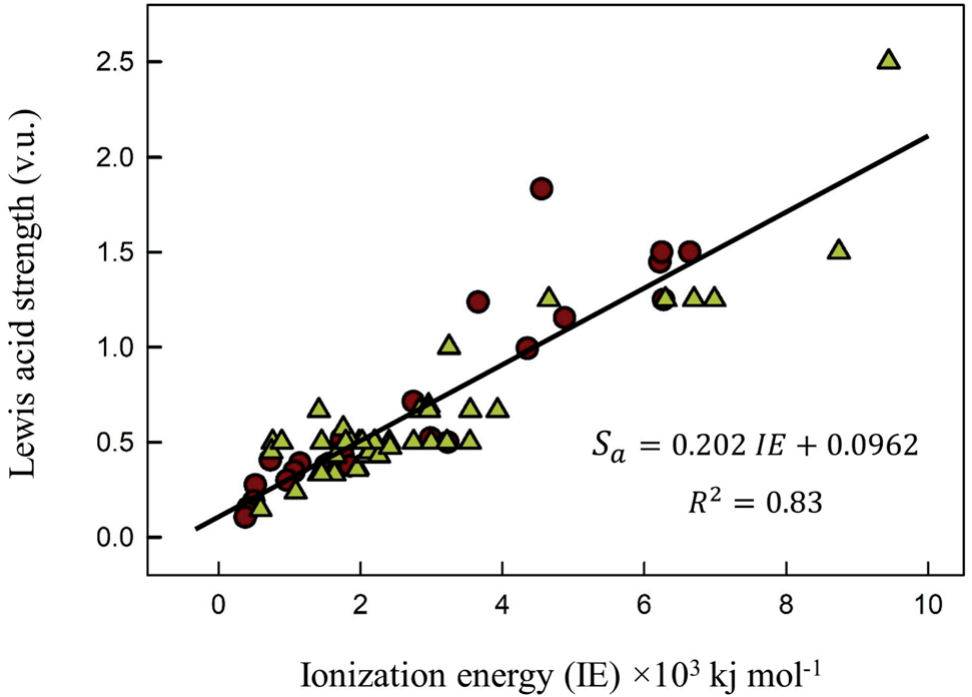
Relation between Lewis acid strength (v.u.) as a function of the *n*^th^ ionization energy of the cation (kJ mol^−1^). Yellow triangles account for less than 10 coordination polyhedra; best fit equation for *n* ≥ 10 is given in text.

In Fig. 1 we show the correlation between Lewis acid strength and the *n*th ionization energy for the 64 cations reported here (*n* < 10 coordination polyhedra marked by triangles). Linear fit (solid line) to all data gives *R*^2^ = 0.83. For *n* ≥ 10 coordination polyhedra (25 cations), the best-fit equation becomes

6 *S*_a_ = 0.315 × IE^0.807^

with *R*^2^ = 0.91. Eqn (6) is very similar to the equation reported by Gagné & Hawthorne for O^2−^ (*S*_a_ = 0.884 × IE^0.807^, *R*^2^ = 0.90).^86^

We also note a high correlation (*R*^2^ = 0.96) between the ratio 
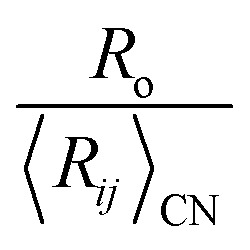
 and Lewis acidity (Table 1)7
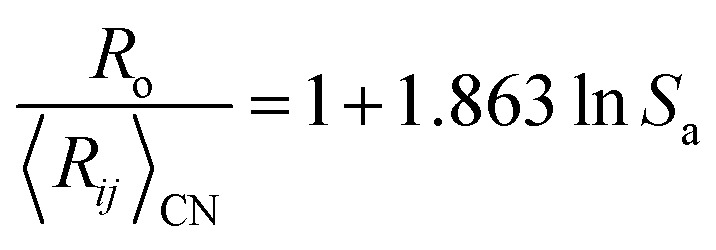
for the 22 cations with *n* ≥ 10 coordination polyhedra for which bond-length data are available to calculate said ratio. This shows strong dependence of bond-valence parameter *R*_o_ to the Lewis acidity of the cation.

## Bond-length variation

We generally observe much narrower bond-length ranges for nitrides than we do for oxides and oxysalts. This observation is likely due to a combination of the nascent sampling of the compositional space of inorganic nitrides and a focus by experimentalists on simple compositions and structures as a result of the significant challenges associated with synthesizing and growing these compounds. In fact, of the 18 transition metal configurations with bond-length range > 0.75 Å when bonded to O^2−^,^58^ only three configurations are observed in our dataset for nitrides: ^[6]^Mn^2+^ (*n* = 6), with bond lengths 2.170–2.417 Å, ^[6]^Cu^2+^ (*n* = 3), with bond lengths 1.879–2.722 Å, and ^[6]^Nb^5+^ (*n* = 2), with bond lengths 2.123–2.129 Å; this compares to bond-length ranges of 0.837, 0.893 and 0.777 Å when bonded to O^2−^,^58^ respectively. Furthermore, the absence of any substantial bond-length variation in many ion configurations bonded to N^3−^ is a result of their observation in (relatively) highly symmetrical structures with inherently little to no variation in *a priori* bond lengths, which was recently demonstrated to be the most common cause of bond-length variation in inorganic solids by Gagné & Hawthorne.^58^

There are 66 cations overlapping this work and that of Gagné & Hawthorne for oxides^58,69–72^ from which we can directly compare mean bond-length and average observed coordination number. We find that cation–N^3−^ mean bond-lengths are on average 0.027 Å longer than their O^2−^ counterparts (0.034 Å when weighting these differences by the number of N^3−^ coordination polyhedra per cation) with mean bond-length differences varying between 0.318 Å shorter (Ag^+^) and 0.196 Å longer (Hf^4+^) when bonded to N^3−^. The average observed coordination number is 1.078 lower for cations bonded to N^3−^*vs.* O^2−^ (1.159, weighted).

Comparing the data on the basis of ion configurations (*i.e.* as a function of oxidation state and coordination number), the average increase in mean bond-length for N^3−^ coordination polyhedra is 0.118 Å (0.106 Å when weighting these differences by the number of N^3−^ coordination polyhedra per cation), over 112 overlapping ion configurations. In comparison, the ionic radii for ^[4]^N^3−^ and ^[4]^O^2−^ are 1.46 and 1.38 Å, respectively.^68^ Thus, our data show a slightly more pronounced increase in mean bond-lengths for N^3−^*vs.* O^2−^ than predicted *via* the addition of ionic radii, with the largest increase for ^[12]^Rb^+^ (0.339 Å) and largest decrease for ^[4]^Ag^+^ (−0.120 Å).

We do not observe structures with N–H⋯N complexes in our neutron diffraction dataset; only N–H⋯O (*n* = 25) and O–H⋯N (*n* = 4). For N–H bonds, the average length is 0.999 Å with a range of 0.915–1.025 Å; for N⋯H bonds, the average length is 2.269 Å, with a range of 2.034–2.610 Å.

### Anomalous bond-length distributions

**Fig. 2:**
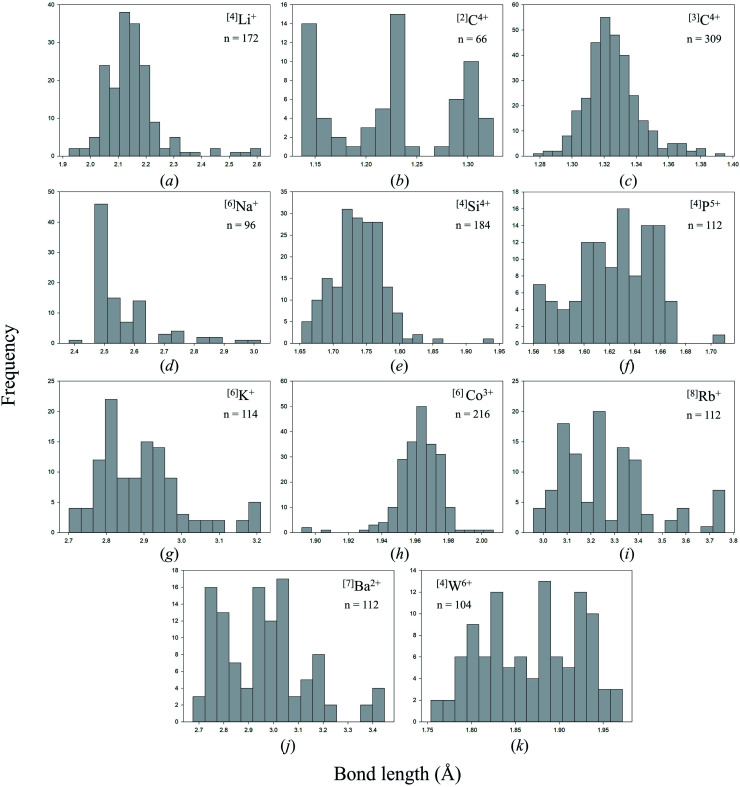
Bond-length distributions for selected ion configurations bonded to N^3−^: (a) ^[4]^Li^+^, (b) ^[2]^C^4+^, (c) ^[3]^C^4+^, (d) ^[6]^Na^+^, (e) ^[4]^Si^4+^, (f) ^[4]^P^5+^, (g) ^[6]^K^+^, (h) ^[6]^Co^3+^, (i) ^[8]^Rb^+^, (j) ^[7]^Ba^2+^, (k) ^[4]^W^6+^.

Bond lengths are expected to form positively skewed Gaussian distributions as a result of the interplay between Coulomb attraction and Born repulsion for ion pairs (Fig. 2c gives such an example for ^[3]^C^4+^ bonded to N^3−^). Gagné & Hawthorne state that deviations from such shape are the result of bond-topological, electronic and/or crystal-structure effects,^58^ which they go on to describe for all cations of the periodic table observed bonding to O^2−^ in inorganic structures.^58,69–72^ Notable examples of anomalous bond-length distributions include a tri-modal distribution for ^[4]^P^5+^–O^2−^ bonds,^70^ caused by varying bond-valence requirements as a function of the degree of polymerization of the [PO_4_]^3−^ oxyanion (*i.e.* bond-topological effects), a tri-modal distribution for ^[6]^Mo^6+^–O^2−^ bonds,^58^ arising from a combination of pseudo Jahn–Teller and bond-topological effects, and anomalously long tails at longer bond lengths for penta-coordinated ion configurations of Cr^2+^, Co^2+^ and Cu^2+^, also caused by pseudo Jahn–Teller and bond-topological effects.^58^

Fig. 2 shows bond-length distributions for the data of Table 2 (cations bonded solely to N^3−^) for *n* > 100 bond lengths and a few more interesting configurations. Due to a general paucity of data, partly due to the difficulty of growing inorganic nitrides as single crystals, few ion configurations have sufficient data for a distinct shape to arise from their bond-length distribution.

Visual inspection of these bond-length distributions shows departure from regular behavior for a handful of ion configurations. For the ^[2]^C^4+^ configuration (Fig. 2c), we observe a tri-modal distribution of bond lengths with peaks at ∼1.15, ∼1.22 and ∼1.31 Å. Converting these bond lengths into bond valences gives 2.62, 2.00 and 1.42 v.u., respectively. Thus, the peak at ∼1.22 Å arises from symmetrical bonds of N=C=N carbodiimide units, such as in the structure of SrCN_2_ (ref. 87) (75040) with *a priori* (observed) bond valences 2 × 2 v.u. (1.933 and 1.985) for C^4+^ (6 × 1/3 v.u. for Sr^2+^) calculated using the method of Gagné & Hawthorne^88^ (Table S2†). The two other peaks of Fig. 2c are complementary, with bond-valence sum ≈ 4 v.u.; they result from N–C≡N cyanamide units, for example in Ag^+^N(CN)_2_ (ref. 89) (843) with *a priori* (observed) bond valences 1.5 (1.524) and 2.5 (2.626) v.u. for C^4+^, and 2 × 0.5 (0.511) v.u. for Ag^+^. Thus, this result shows that bond valences are not perfectly distributed into a [3 + 1] v.u. arrangement in cyanamide units; a bond-valence of 3 v.u. would require a bond length of 1.115 Å.

**Fig. 3:**
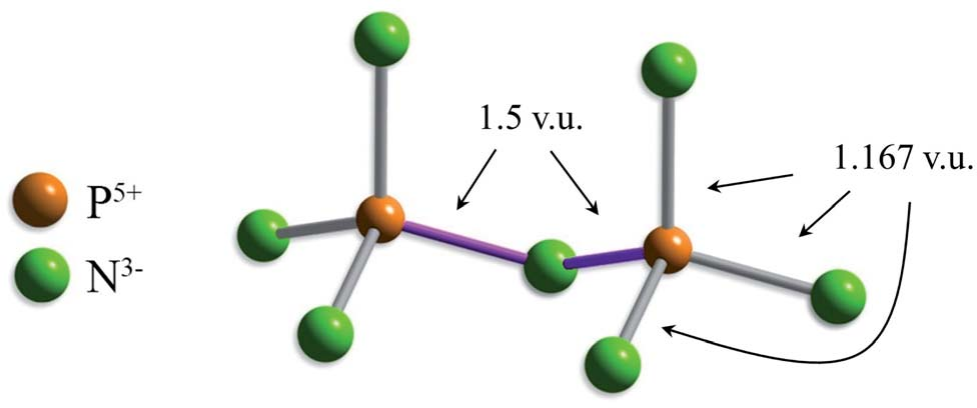
Unequal distribution of bond valences required by the valence-sum rule, shown for a corner-sharing dimer (3 v.u. for N^3−^, green; 5 v.u. for P^5+^, orange); even distribution of bond valences (4 × 1.25 v.u.) would leave the bridging ^[2]^N^3−^ ion under-bonded by 0.5 v.u.

Two other bond-length distributions are observed with slight irregularities; those of ^[4]^P^5+^, and ^[4]^W^6+^ (Fig. 2f and k, respectively). In both instances, the root cause for irregularity is non-local bond-topological asymmetry. For these ion configurations, competition between the bond-valence constraints of the cation and its bonded anions requires uneven distribution of bond valences (thus bond lengths; eqn (1)) in cation and/or anion coordination polyhedra. This mechanism of bond-length variation was described for ^[4]^P^5+^ bonded to O^2−^ by Gagné & Hawthorne,^70^ and was later extended to transition metals and described under the umbrella of *bond-topological effects*.^58^ For example, ^[4]^P^5+^ ideally forms 4 bonds that are 1.25 v.u. in strength; however, the ideal bond strengths for a bridging ^[2]^N^3−^ ion are 2 × 1.5. v.u. Thus, for polymerization into corner-sharing dimers, ^[4]^P^5+^ adapts to the bond-valence requirement of ^[2]^N^3−^ and increase the strength of one bond to 1.5 v.u., weakening the three other bonds to 1.167 v.u (Fig. 3). For a chain of corner-sharing tetrahedra, ^[4]^P^5+^ adjusts to 2 × 1.5 and 2 × 1 v.u.—so and so forth for different combinations of degrees of polymerization, number of shared vertices, and varying coordination number of the bridging anion(s). These different combinations result in multiple maxima in the bond-length distribution of cations prone to bond-topological effects; full rationalization of the shape of their bond-length distributions is achieved *via* the calculation of *a priori* bond valences for their constituent crystal structures. In our dataset, ^[4]^P^5+^ and ^[4]^W^6+^ are observed to polymerize into oligomers, chains, rings, sheets, clusters and frameworks, and we do not resolve the bond-valence requirements of each scenario here.

While other apparent instances of multi-modality observed in Fig. 2 result from paucity of data, our full dataset abounds with subtle bonding irregularities that allow a glimpse into the intriguing bonding properties of nitrides *sensu lato*, of which many bear technological importance. In the next part of this work, we examine structural and electronic effects associated with anomalous bond lengths in our dataset, thus shifting our focus toward uncovering less traditional bonding properties of inorganic nitrides that offer promising opportunities in the exploration of their bulk compositional space.

## Opportunities for bulk exploratory synthesis

### On the exploration of new compositional spaces

This past decade has seen a rapid increase in the development of high-throughput (HT) computational methods applied to materials discovery. These methods, usually rooted in Density Functional Theory (DFT) and/or Machine Learning (ML), allow screening of large compositional spaces in search of *yet-to-be-observed* compounds with desired functional properties. A growing number of HT studies include experimentally validated predictions (see a list compiled by Jain *et al.* for DFT^90^),^28,91–96^ partly owing to their suitability to *synthesizability analysis*^3,28,97–99^—an emerging concept used to curb the difficulty of predicting the outcome of chemical reactions in the solid state.

Today, rapid increase in available computational power has by-and-large transformed the problem of *in silico* exploration from one of computational feasibility to one of *a priori* identifying compositional spaces of interest^100,101^—a pursuit that has historically been realized *via* rules of crystal chemistry^102,103^ and heuristic concepts. However, several issues cloud the exploration of new compositional spaces *via* DFT approaches, separate from the difficulties traditionally associated with the theory itself (*e.g.* strongly correlated systems, substitutional disorder, ground state calculations that ignore a potentially non-negligible entropic contribution to structure stability,^104^*etc.*). For example, while exploration of extensive compositional spaces is currently manageable for ternary phases (as most recently evidenced by the work of Sun *et al.*^28^), quaternary-and-higher compositional spaces are exponentially more computationally expensive for they require their stability be evaluated against an exponentially large number of stoichiometrically feasible phases of lower order. Current approaches are further limited to a relatively small set of known elementary, binary, and ternary crystal structures which may not necessarily conform to the chemical compositions investigated. Nonetheless, these problems are largely temporal, and should progressively lessen with an ever-increasing supply of computational power and the incremental discovery of new crystal structures.

A more difficult (yet hardly discussed) problem is the fundamental inability of DFT to model energetically degenerate and pseudo-degenerate electronic states, whereby vibronic coupling giving rise to Jahn–Teller (and pseudo Jahn–Teller) distortions do not conform to the Born–Oppenheimer and adiabatic approximations underpinning DFT.^105,106^ This is problematic with regard to the startling extent to which vibronic coupling occurs in inorganic solids, as recently shown by Gagné & Hawthorne *via* large-scale bond-length dispersion analysis for oxides and oxysalts;^58^ of 147 configurations of transition metal ions observed bonding to O^2−^, 52 configurations were observed with anomalous bond-length distributions, 46 of which partially or entirely due to vibronic coupling. Pending development of vibronic patches to DFT methods for HT investigation (*e.g.* ref. 107) and widescale evaluation of their efficacy, complementing DFT calculations with different approaches could possibly attenuate the vibronic coupling problem; for example, prediction of non-centrosymmetric behavior is within the purview of machine learning, which has been used in combination with DFT calculations to insulate promising non-centrosymmetric compositions from large compositional spaces (*e.g.* Ruddlesden–Popper oxides^108^). The practice of combining DFT and machine learning approaches was recently reviewed by Schleder *et al.*^109^

Notwithstanding the above, HT computing should not be mistaken for a one-stop solution to the multi-faceted issue of exploratory synthesis.^110^ While HT methods play a critical role in fast-tracking materials discovery *via* the identification of “missing compounds” and the calculation of their properties,^28,100^ they are limited to rehashing data derived from known chemical spaces; navigating the totality of all existing compositional and structural spaces is categorically intractable for state-of-the-art HT methods.^111^ Thus, the discovery of new compositional spaces and of next-generation materials largely continues to lie in the ingenuity of the crystal and synthetic chemists. This assertion is particularly relevant to the exploration of inorganic nitrides, whose remarkable range of metastability suggests an exceptionally broad spectrum of observable compositions.^97^ Combined with the main takeaways of our bond-length dispersion analysis, this proposition leads us to affirm that the chemical potential of inorganic nitrides has barely been scratched, thus pressing the need for exploration *outside* known compositional spaces.

Below, we use the systematic nature of our bond-length dispersion analysis to identify various functional building blocks for integration into new compositions and crystal structures. Such an approach has disproportionate potential for leading to new disruptive compounds, and could advantageously be integrated into HT approaches to *a priori* identify areas of interest *in lieu* of expanding resources in low-return chemical spaces. Further integration of the data from Tables 1 and 2 may be used to rapidly verify the structural validity of these modeled compounds before expanding resources on them.

### New compositional spaces for the exploratory synthesis of inorganic nitrides

In light of the recent extensive review of the crystal chemistry of oxides and oxysalts by Gagné & Hawthorne,^58,69–72^ we investigate our dataset of inorganic nitride structures for the occurrence of bond topological, electronic and/or crystal-structure effects, placing emphasis on the functional properties resulting from these effects. Compositional and structural divergences are expected between the compounds making up these two datasets. For example, lower cation coordination numbers are expected in inorganic nitrides due to the larger size of N^3−^*vs.* O^2−^ (as evidenced by our comparison of mean bond lengths between N^3−^ and O^2−^ structures, above). We further expect (and observe) similar or lower oxidation states for cations in inorganic nitrides since O^2−^ is better at stabilizing high metal oxidation states as a result of its higher electronegativity (3.44 *vs.* 3.04 for N), or alternatively, higher electron affinity.^112^ Furthermore, the reduced ionicity of the chemical bonds made by N^3−^ allows formation of exceptionally strong and localized bonds that can lock-in energetically unfavorable atomic arrangements;^97^ this is particularly relevant to electronegative p-block and d-block elements.

In addition to our principal dataset, we surveyed the chemical behavior of inorganic nitrides in coordination complexes; many molecular features of coordination complexes are preserved as they incorporate into crystal structures, and the carrying of their electronic properties is often more important than the steric constraints of space-group symmetry and long-range order.^113^ A chemically intuitive treatment of chemical bonding thus follows from the common simplifying assumption of no translational symmetry,^114,115^ allowing the study of molecular fragments *via* ligand field theory; the slight loss in accuracy (effectively, losing information on “additional” bonding schemes that arise in extended solids as a result of electron delocalization) is greatly overcome by substantial gains in chemical intuition at a local scale, resulting in transferable insight useful to the exploration of new compositional spaces. As such, bonding knowledge derived in coordination chemistry poses as powerful inspiration to solid-state syntheses.^116^

We organize our discussion into five *phenomenological* compositional spaces holding promise for the exploratory synthesis of bulk functional inorganic nitrides (summarized in Table 3), and encourage expansion of this kind of analysis to different chemical subfields whose functional units may differ in composition. In contrast to HT methods, the intent of the following subsections is not to identify specific chemical compositions and/or crystal structures, but to unearth new and promising compositional spaces from an otherwise intractable combinatorial space of chemistries to be used as starting points for more concrete computational and/or synthetic investigations.

**Table 3:** Promising ions for the exploratory synthesis of functional inorganic nitrides and comparison to their oxide counterparts

Confirmed (O^2−^)^58,69–72^	Confirmed (N^3−^)	To be investigated (N^3−^)^a^	Relation to O^2−^
**Full-fledged multiple-bond formation (>1.95 v.u.)**			
^[3]^N^5+^, ^[4]^Cl^7+^, ^[4]^V^5+^, ^[4]^Cr^6+^, ^[5,6]^Mo^5+^, ^[4–6]^Mo^6+^, ^[6]^W^6+^, ^[4–5]^Re^7+^, ^[4–5]^Os^8+^, ^[6–8]^U^6+^	^[2]^C^4+^, ^[2]^N^5+^, ^[2]^S^4+^, ^[5]^Mo^6+^, ^[4]^W^6+^	V^4+^, V^5+^, Cr^5,6+^, Nb^5+^, Mo^5+^, Tc^7+^, Ta^5+^, Re^7+^, Os^8+^, U^6+^	*χ*_N^3−^_ < *χ*_O^2−^_; multiple-bond formation facilitated for N^3−^. Triple bond impossible for O^2−^; special geometry required for N^3−^

		(>1 v.u.: Ti^4+^, Se^4,6+^, As^5+^, Br^5,7+^, Te^4,6+^, I^5,7+^, W^5+^, Os^6,7+^, Bi^5+^, Np^5,6,7+^)	

**Coupled electronic-vibrational degeneracy: the Jahn–Teller effect**			
^[6]^Cr^2+^, ^[6]^Mn^3+^, ^[6]^Cu^2+^	^[3]^Cr^3+^, ^[6]^Co^3+^, ^[6]^Cu^2+^	^[6]^V^3+^, ^[6]^Cr^2+^, ^[6]^Mn^3+^, ^[6]^Fe^2+^, ^[3]^Fe^3+^, ^[6]^Co^2+^, ^[6]^Mo^5+^, ^[6]^Os^7+^	*χ*_N^3−^_ < *χ*_O^2−^_; increased covalency thus distortion magnitude for N^3−^

Minor: ^[6]^Co^2+^, ^[6]^V^3+^ and ^[6]^Mo^5+^, ^[6]^Os^7+^			

**Coupled electronic-vibrational near-degeneracy: the pseudo Jahn–Teller effect**			
^[4–7]^Ti^4+^, ^[4–6]^V^5+^, ^[6–10,12]^Y^3+^, ^[6–10]^Zr^4+^, ^[4–8]^Nb^5+^, ^[4–6]^Mo^6+^, ^[6–8]^Hf^4+^, ^[6–7]^Ta^5+^, ^[4–6]^W^6+^, ^[4–6]^Re^7+^, ^[4–6]^Os^8+^. Minor: ^[6–8]^Sc^3+^, ^[5–6]^V^4+^, ^[4]^Cr^6+^, ^[4]^Mn^7+^, ^[6–10,12]^Y^3+^; ^[5]^Cr^2+^, ^[5]^Co^2+^, ^[5]^Cu^2+^, ^[6]^Zn^2+^, ^[6]^Cd^2+^, ^[6]^Hg^2+^	^[6]^Y^3+^, ^[4]^Mo^6+^, ^[8]^Hf^4+^, ^[4]^Ta^5+^, ^[4]^W^6+^	Sc^3+^, Ti^4+^, Zr^4+^	*χ*_N^3−^_ < *χ*_O^2−^_; optimal match of atomic orbital energies shifted to cations of lower electronegativity. Increased covalency thus distortion magnitude for N^3−^

		Observed, but distortion magnitude inconclusive due to paucity of data: ^[4]^V^5+^, ^[4]^Cr^6+^, ^[4,6]^Nb^5+^	

**Lone-pair stereoactivity**			
^[3]^P^3+^, ^[3]^S^4+^, ^[2,4]^Cl^3+^, ^[3]^Cl^5+^, ^[3–8]^As^3+^, ^[4–10]^Se^4+^, ^[6–8]^Br^5+^, ^[3–9]^Sn^2+^, ^[3–9]^Sb^3+^, ^[3–12]^Te^4+^, ^[6–9]^I^5+^	^[6–8]^Tl^+^, ^[3]^Sn^2+^, ^[5,7]^Pb^2+^	Sn^2+^, As^3+^, Sb^3+^, Se^4+^; possibly In^+^, Ge^2+^, Te^4+^, Bi^3+^	*χ*_N^3−^_ < *χ*_O^2−^_; optimal match of atomic orbital energies shifted to cations of lower electronegativity

Minor: ^[3–12]^Tl^+^, ^[3–12]^Pb^2+^, ^[3–10,12]^Bi^3+^			

**Polymerization:** ^**[4]**^ **M** ^**6+**^ **configuration**			
^[4]^Si^4+^, ^[4]^Ge^4+^, ^[4]^Ti^4+^, ^[4]^Cr^4+^	^[4]^Mo^6+^, ^[4]^W^6+^	^[4]^S^6+^, ^[4]^Se^6+^, ^[4]^Cr^6+^	Cation OS/CN ratio of 1.5 required for prolific polymerization *via*^[2]^N^3−^, *vs.* a ratio of 1 for ^[2]^O^2−^

a Includes cations of high oxidation state (OS) that may be challenging to achieve experimentally.

### Multiple-bond formation

One of the primary causes of bond-length variation in inorganic solids is the formation of “multiple bonds” between ion pairs.^58^ Inorganic nitrides are exciting materials for in-depth study of this phenomenon and its ensuing properties, for they are a rare class of solid-state compounds where triple-bond formation (*i.e.* up to 3 v.u. in strength) is possible. In inorganic nitrides, multiple-bond formation is commonly observed for metal nitrido complexes, *i.e.* coordination complexes which contain one or more atoms of nitrogen bound only to transition metals. In these complexes, multiple-bond formation (commonly called “double” or “triple” bonds) occurs *via* mixing of anion p orbitals and unfilled metal d and/or f orbitals.^117,118^

**Fig. 4:**
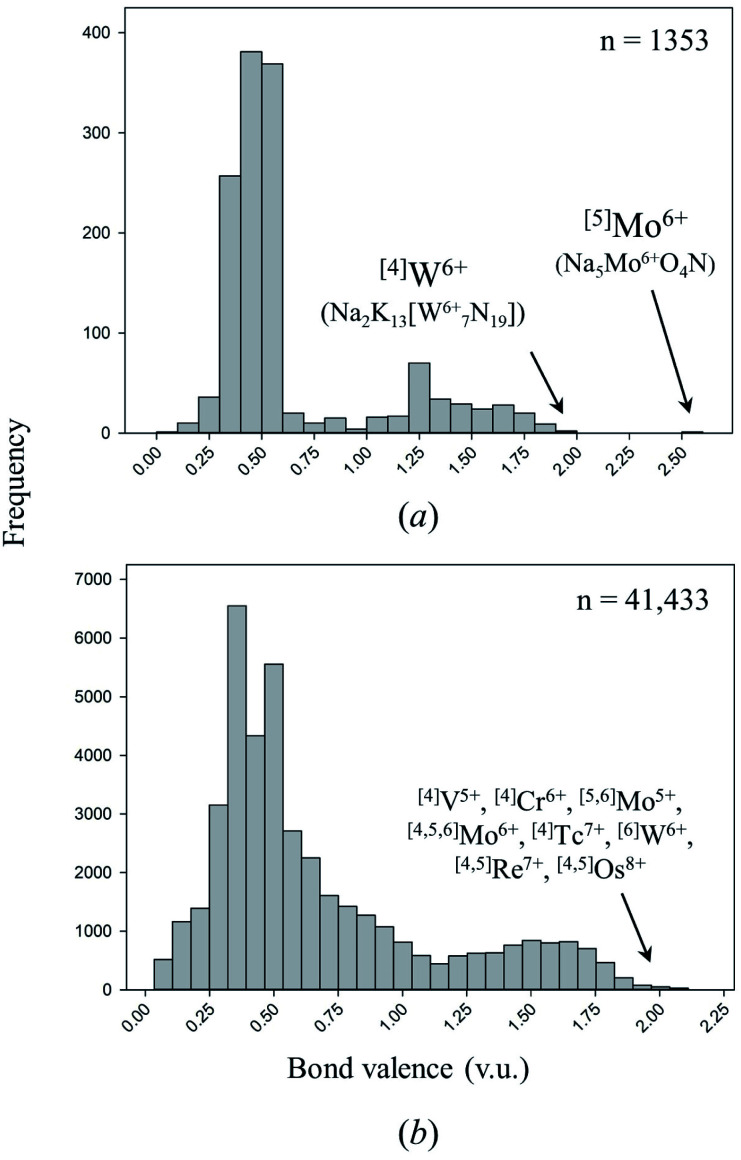
Distribution of bond valences for all transition-metal configurations observed in (a) this work, (b) the dataset of Gagné & Hawthorne for transition metals bonded to O^2−^.

Coordination compounds containing nitrido complexes are ubiquitous in chemical literature, largely driven by the desire to improve our understanding of the mechanism of nitrogen fixation (which is assumed to undergo nitrido-complex formation).^119^ Comprehensive reviews bring into light the remarkable chemical versatility of doubly- and triply-bonded N^3−^.^120–124^ Despite this wealth of information, very few compounds containing double or triple M–N bonds have been synthesized and characterized in the solid state. Of the 137 cation configurations we observe bonded solely to N^3−^ (Table 2), only four configurations are observed with one or more structures containing a bond valence > 1.95 v.u.; three are non-metals, the other is ^[4]^W^6+^ (including oxynitrides only adds one configuration to this tally: ^[5]^Mo^6+^). In comparison, Gagné & Hawthorne report 17 of 461 cation configurations with bond valence > 1.95 v.u. (*i.e.* discrete terminal double bonds to O^2−^) in oxide and oxysalt structures, 12 of which for transition metals (Fig. 4).^58^ Although inorganic nitrides have the ability to make terminal bonds up to 3 v.u. in strength, we observe no such bonds in our dataset. The strongest metal bond observed is for ^[5]^Mo^6+^ in oxynitride Na_5_Mo^6+^O_4_N^125^ (55113) with a Mo^6+^–N^3−^ distance of 1.719 Å, representing a bond valence of 2.583 v.u.

Evidently, the potential for making strong terminal bonds to N^3−^ is untapped; combined with the incredible variety of known nitrido complexes spanning most metals of the periodic table (including U^118,126^), doubly and triply-bonded inorganic nitrides are promising candidates for exploratory synthesis. Such compounds may not only be helpful in clarifying the mechanism of N_2_ reduction, but could possibly be used as heterogeneous catalysts in the development of a post-Haber–Bosch process proceeding at milder reaction conditions; the remarkable diversity of known nitrido-complex compounds shows considerable promise for fine tuning the kinetics of N_2_ and H_2_ activation. Metal-nitrido coordination complexes have also been reported as a key component to the catalytic oxidation of water,^127^ alkanes,^128^ alkenes and alcohols,^129^ thus showing further promise for their use as solid-state catalysts.

There are 66 of 76 cations and 112 of 137 cation configurations overlapping this work and that of Gagné & Hawthorne for oxides and oxysalts,^58,69–72^ whereby non-overlapping configurations primarily result from the preference of inorganic nitrides for lower cation oxidation states and/or coordination numbers. Based on the similar crystal-chemical behavior of these classes of compounds, their significant overlap in cation configurations, and the isoelectronic nature of N^3−^ and O^2−^, we assume that cation configurations yet-to-be-observed in inorganic nitrides with equal or lower oxidation states and/or coordination numbers than their oxide and oxysalt counterparts either result from challenging synthesis or lack of synthetic attempts. Comparing the dataset of Gagné & Hawthorne against the present work for cation configurations with bonds > 1 v.u., we conclude that promising opportunities for multiple bond-formation (1–3 v.u.) in inorganic nitrides include cations such as Ti^4+^, V^4+^, As^5+^, Se^4,6+^, Br^5+,7+^, Mo^5+^, Tc^7+^, Te^4,6+^, I^5,7+^, W^5+^, Re^7+^, Os^6,7,8+^, Bi^5+^, U^6+^ and Np^5,6,7+^; further syntheses are also warranted for V^5+^, Cr^5,6+^, Nb^5+^, Mo^6+^, Ta^5+^ and W^6+^.

### Coupled electronic-vibrational degeneracy: the Jahn–Teller effect

The Jahn–Teller effect (JTE) is a mechanism of symmetry-breaking that results from strong electron–vibrational (vibronic) and electron–phonon interactions in molecules and crystals, respectively,^130^ and is characterized by energetically favorable occupancy of electronic states ensuing degeneracy breaking polyhedral distortion. Although the JTE is commonly observed in coordination complexes of N^3−^ (including nitrido complexes,^131^ which are for example used as single-molecule magnets^132^), research into the material properties resulting from the JTE has historically focused on oxide and oxysalt compounds, including colossal magnetoresistance,^133^ superconductivity,^134^ improved electrochemical performance of cathode materials (*via* the “opening” of diffusion channels),^135,136^ and magnetic-dielectric bistability.^106^

Gagné & Hawthorne's recent bond-length dispersion analysis for transition metals bonded to O^2−^ identifies 4 out of 52 typically highly distorted ion configurations where coupled electronic-vibrational degeneracy is the principal underlying cause of bond-length variation, ^[6]^Cr^2+^, ^[6]^Mn^3+^, ^[6]^Cu^2+^ and ^[6]^Os^7+^, and an additional three configurations where it is a minor contributing factor: ^[6]^Co^2+^, ^[6]^V^3+^ and ^[6]^Mo^5+^. Considering the lower electronegativity of N *vs.* O (which entails more covalent bonds to transition metals) and the isoelectronic nature of these ligands, there is reason to believe that the above ion configurations (and possibly others) may experience the JTE with larger distortion magnitude when bonded to N^3−^.

In the present dataset, we observe three cation configurations (bonded solely to N^3−^) for which anomalous bond lengths can be attributed to the JTE: ^[3]^Cr^3+^, ^[6]^Co^3+^ (weak) and ^[6]^Cu^2+^ (strong). Ion configurations ^[6]^Mn^3+^, ^[6]^Os^7+^ and ^[6]^Mo^5+^ have yet to be observed, while ^[6]^Cr^2+^ and ^[6]^V^3+^ are each observed in one structure, refined as regular octahedra, and ^[6]^Co^2+^ observed in two structures, also refined as regular octahedra. ^[3]^Cr^3+^ is observed in Ca_3_Cr^3+^N_3_ (40205) with bond lengths 2 × 1.766 and 1.863 Å.^137^^[6]^Co^3+^ ([Ar]3d^6^) is observed in (NH_3_)_5_Co^3+^NCSCo^3+^(CN)_5_H_2_O (4094), with two anomalously short bond lengths of 1.907 and 1.932 Å in *trans* configuration, and 4 equatorial bonds ranging 1.972–1.977 Å,^138^ and in (Co^3+^(NH_3_)_6_)(Np^5+^O_2_(C_2_O_4_)_2_)(H_2_O)_1.5_ (155531) with two short bond lengths of 1.892 and 1.894 Å in *trans* configuration and 4 equatorial bonds ranging 1.937–1.970 Å (spin unknown in both structures).^139^^[6]^Cu^2+^ ([Ar]3d^9^) is observed in three structures bonded solely to N^3−^, and ten structures in mixed proportions with O^2−^. The Jahn–Teller effect is observed in all cases, to a largely varying extent, and where the two weakest bonds (<0.2 v.u.) are always to O^2−^. For the three Cu^2+^N_6_ octahedra, the observed bond lengths are 4 × 2.061 and 2 × 2.612 Å for Cu(NH_3_)_4_)(Ag(SCN)_3_ (10065),^140^ 4 × 1.997 and 2 × 2.369 Å in Cu[B(CN)_4_]_2_ (415546),^141^ and 2 × 1.879, 2 × 1.965 and 2 × 2.722 Å for K_2_CuFe(CN)_6_ (99499).^142^

The surprisingly large range of bond lengths observed for the latter structure, 0.843 Å, appears to support the case for higher distortion magnitude in inorganic nitrides; of 365 polyhedra observed bonding to O^2−^ by Gagné & Hawthorne,^58^ only one is observed with a larger range of bond lengths, 0.870 Å, for mrázekite Bi_2_^3+^Cu_3_^2+^(OH)_2_O_2_(PO_4_)_2_(H_2_O)_2_ (71934).^143^

**Fig. 5:**
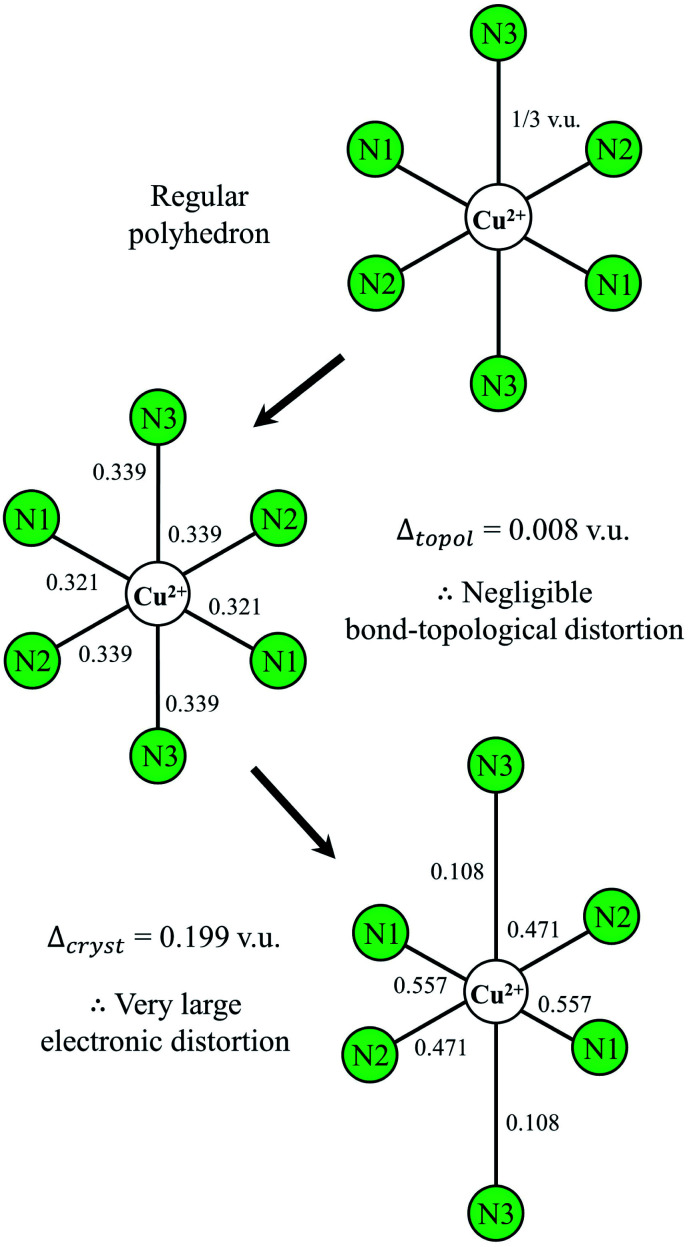
Effect of bond-topological and electronic effects on polyhedral distortion for the Cu^2+^N_6_ octahedron in K_2_CuFe(CN)_6_. All values are in valence units.

To verify that the large variation of bond lengths is not due (or partly due) to non-local bond-topological asymmetry in K_2_CuFe(CN)_6_, we calculate values of Δ_topol_ and *Δ*_cryst_ for the Cu^2+^N_6_ octahedron using the method of Gagné & Hawthorne;^58^*Δ*_topol_ is calculated as the mean (absolute) weighted deviation between the bond valences of a given polyhedron and that of its regular variety with equidistant bond lengths, *i.e.* its Pauling bond strength, and *Δ*_cryst_ is calculated as the mean (absolute) weighted deviation between the *a priori* and observed bond valences—thus encompassing all effects not arising from bond-topological arguments. We calculate *Δ*_topol_ = 0.008 and *Δ*_cryst_ = 0.199 v.u. for the Cu^2+^N_6_ octahedron (Fig. 5); these values clearly indicate that bond-length variation is overwhelmingly due to the JTE, supporting the suggestion of larger distortion magnitude for cations bonded to N^3−^ (*a priori* bond valences are given in Table S2†). The untapped potential for highly distorted Cu^2+^ polyhedra (and other JT-active cations) may have important implications in the design of oxynitride and/or nitride counterparts to cuprate superconductors, with a handful of layered oxynitride^144^ and nitride superconductors already known for d^0^ transition metals.^145,146^

Our dataset contains two cations that seem to be JT-inactive in octahedral coordination to N^3−^ with d^4^ and d^7^ electronic configurations: ^[6]^Cr^2+^ and ^[6]^Co^2+^. ^[6]^Cr^2+^ ([Ar]d^4^, *n* = 1) is observed in [Cr^2+^(NH_3_)_6_]I_2_ (78860)^147^ with reported high spin configuration, refined as a regular octahedron. ^[6]^Co^2+^ ([Ar]d^7^, *n* = 2) is observed in [Co^2+^(NH_3_)_6_](PF_6_)_2_ (30704; spin unknown)^148^ with bond lengths 6 × 2.186 Å, nearly identical to those of [Co^2+^(NH_3_)_6_]Br_2_ (78864)^147^ (6 × 2.176 Å) with Co^2+^ in high spin configuration. In comparison, both ^[6]^Cr^2+^ and ^[6]^Co^2+^ are dominatingly high spin in oxides,^58^ making ^[6]^Cr^2+^ strongly JT-active (with clear-cut bi-modal bond-length distribution) and ^[6]^Co^2+^ weakly JT-active (unimodal bond-length distribution). It is possible that the observation of regular Co^2+^/Cr^2+^N_6_ octahedra is due to the dynamic JTE, which was shown to be present in Co^2+^N_6_ terpyridine complexes by Kremer *et al.*^149^ In oxynitride Co^2+^(H_2_O)_2_Ni^2+^(CN)_4_·4H_2_O (59366; spin unknown),^150^ bond distances are 2 × 2.095 and 2 × 2.101 Å to N^3−^, and 2 × 2.128 Å to O^2−^ (0.380, 0.376 and 0.319 v.u. respectively); in Cs_2_Co^2+^(S_8_(Re^3+^(CN))_6_)(H_2_O)_2_ (89491),^151^ bond distances are 2.052 (0.390) and 2.092 (0.351) to O^2−^, and 4 × 2.161 Å (0.332 v.u.) to N^3−^, indicating that ^[6]^Co^2+^ may be JT-active when bonded to a mixture of O^2−^ and N^3−^.

Other cations prone to the JTE and warranting further investigation include Fe^2+^, Co^3+^, V^3+^ and Mo^5+^ in octahedral coordination (weak); the observation of ^[6]^Mn^3+^ (strong) seems less likely as Mn^3+^ appears to prefer lower coordination numbers when bonded to N^3−^. Cations with lower coordination numbers (*e.g.*^[3]^Fe^3+^ (ref. 152)) do present further opportunity to study the JTE in inorganic nitrides, although bond-length variations associated with lower coordination numbers are usually modest.

### Coupled electronic-vibrational near-degeneracy: the pseudo Jahn–Teller effect

The pseudo Jahn–Teller effect (PJTE) is a mechanism of symmetry breaking that results from the vibronic mixing of (two or more) electronic states sufficiently close in energy to interact under nuclear displacement.^130,153^ Vibronic mixing usually (but not necessarily) occurs between the highest occupied molecular orbital (HOMO) and the lowest unoccupied molecular orbital (LUMO), with a distortion mode having the same symmetry as the HOMO to LUMO transition.^153^ Although occurrence of the PJTE is not encumbered by *a priori* limitations such as electronic configuration, the PJTE is primarily observed for d^0^ transition metals, with resulting non-centrosymmetric behavior responsible for a host of technologically relevant properties including ferroelectricity,^59,154^ ferromagnetism,^155^ multiferroicity,^156^ piezoelectricity,^59^ photocatalysis,^157^ nonlinear optics,^59,63^ magnetic-dielectric bistability,^106^*etc.* The PJTE is of particular interest to materials design, for the structural instability resulting from this effect can be controlled by means of electronic rearrangements induced by redox processes, electromagnetic fields, external pressure, and more,^153^ with potential applications spanning bistable atomic switches,^158–160^ and control over sorption characteristics of catalysts to move beyond the Sabatier principle.^161^

Because vibronic phenomena do not conform to the Born–Oppenheimer and adiabatic approximations, the modeling of JTE- and PJTE-active compounds is ill-suited to DFT investigation and requires careful treatment that precludes high-throughput analysis.^106^ For this reason, the derivation of empirical trends and use of heuristic concepts is particularly important to the exploration of this phenomenon. Gagné & Hawthorne recently showed that the PJTE is the 2^nd^ most frequently encountered cause of bond-length variation in transition metals when bonded to O^2−^ (after bond-topological effects).^58^ In their work, the PJTE is observed as the main reason underlying anomalous bond-length distributions (in terms of shape and/or range) for 29 of 52 transition metal configurations, in addition to 11 ion configurations for which it is present in minor yet significant ways, covering electronic configurations d^0^, d^4^, d^7^, d^9^ and d^10^, and coordination numbers [4]–[10]. Because studies covering PJTE-active cations have almost exclusively focused on oxides and oxysalts, our understanding of this phenomenon is particularly lagging in inorganic nitrides, where few syntheses incorporating d^0^ transition metals have been attempted (with the exception of the group of Kazuhiko Maeda who studied a handful of oxynitrides with d^0^ transition metals as photocatalytic materials^162^).

Because PJTE instabilities can be of any symmetry within the group representation (thus are indiscriminate of coordination number), occurrence of the PJTE can be reduced to a problem of energy gap between interacting electronic states. In their study of octahedrally coordinated d^0^ transition metals, Kunz & Brown^163^ observed an increasing degree of distortion with decreasing HOMO–LUMO gap, which in turn correlates to the size and charge of the d^0^ cation (this trend was later described in terms of electronegativity,^164^ whereby more electronegative cations lead to larger distortions when bonded to O^2−^). While similar behavior is expected for nitrides, the lower electronegativity of N (3.04) compared to O (3.44) entails a shift of the electronic states of N^3−^ to higher energies, thus affecting the HOMO–LUMO gap (and distortion magnitude) compared to O^2−^. As a result, the occurrence of PJT-driven distortion for bonds made to N^3−^ are shifted to cations of lower electronegativity in relation to O^2−^ for a better match of orbital energies. In addition, the lower electronegativity of N (closer to that of transition metals) entails potential for larger distortion magnitude (measured as *Δ*_cryst_) owing to increased covalency of the M–N bond. However, others factors (*e.g.* nearest-neighbor identity^153,164^) may have an overwhelming effect on the energy gap of the interacting states. While rationalizing the occurrence of the PJTE *via* quantum mechanical arguments is now commonplace, predicting the occurrence of a PJT distortion from simple crystal-chemical principles remains an open problem.

Table 4 summarizes bond-length information for ion configurations prone to the PJTE overlapping the present dataset and that of Gagné & Hawthorne for oxides and oxysalts.^58^ Italicised entries are for ion configurations where the PJTE is a minor contributor to bond-length variation in oxides and oxysalts; for example, the effect of polymerization on bond length variation is much larger than that of the PJTE for ^[4]^V^5+^ when bonded to O^2−^. To precisely evaluate the relative distortion magnitude for cations bonded to N^3−^*vs.* O^2−^, one would ideally quantify the proportion of bond-length variation due to the PJTE *via* the method of Gagné & Hawthorne,^58^*i.e.*, by removing the bond-topological contribution to bond-length variation (example given for Cu^2+^ above). Unfortunately, too few crystal structures are available to comprehensively calculate *Δ*_topol_ and *Δ*_cryst_ indices in inorganic nitrides. However, the few crystal structures suited to the calculation of these indices are in support of a shift of the occurrence of the PJTE to cations of lower electronegativity, and hint at larger distortion magnitude for the same cation configurations (data compared to Table S2† of Gagné & Hawthorne^58^). For example, *Δ*_topol_ = 0 and *Δ*_cryst_ = 0.177 v.u. for ^[8]^Hf^4+^ in Hf_3_^4+^N_4_ (97997),^165^ with bond-length range 0.295 Å; in Li_4_(Ta^5+^N_3_) (412585),^166^*Δ*_topol_ = 0.083 and *Δ*_cryst_ = 0.084 v.u. for ^[4]^Ta^5+^, with bond-length range 0.106 Å. Distortion magnitudes attributable to the PJTE are still large for higher-electronegativity transition metals: in LiBa_4_(Mo_2_^6+^N_7_) (74822),^167^*Δ*_topol_ = 0.079 and 0.095 and *Δ*_cryst_ = 0.230 and 0.123 v.u. for two crystallographically distinct ^[4]^Mo^6+^ sites, with bond-length ranges 0.149 and 0.111 Å, respectively; in LiBa_4_(W_2_^6+^N_7_) (74823),^167^*Δ*_topol_ = 0.081 and 0.091 and *Δ*_cryst_ = 0.101 and 0.064 v.u. for two crystallographically distinct ^[4]^W^6+^ sites, with bond-length ranges 0.138 and 0.110 Å, respectively (all *a priori* bond valences in Table S2† herein).

**Table 4:** Comparison of mean bond-length ranges for PJTE-active cations bonded to N^3−^*vs.* O^2−^

Ion configuration	Electronic configuration	Sample size bonded to N^3−^/O^2−^a	Mean bond-length range bonded to N^3−^/O^2−^a (Å)
^*[4]*^ *V* ^*5+*^	d^0^	1/345	0.038/0.118
^*[4]*^ *Cr* ^*6+*^	d^0^	2/169	0.024/0.120
^[6]^Zn^2+^	d^10^	1/193	0/0.169
^[6]^Y^3+^	d^0^	5/25	0.096/0.081
^[4]^Nb^5+^	d^0^	10/2	0.049/0.117
^[6]^Nb^5+^	d^0^	2/240	0/0.290
^*[4]*^ *Mo* ^*6+*^	d^0^	11/434	0.068/0.069
^*[6]*^ *Cd* ^*2+*^	d^10^	4/135	0.067/0.140
^[8]^Hf^4+^	d^0^	1/7	0.295/0.163
^[4]^Ta^5+^	d^0^	5/0^b^	0.0464/—
^[4]^W^6+^	d^0^	26/35	0.124/0.053

a Oxide data taken from Gagné & Hawthorne.^58^

b Not observed bonded to O^2−^.

Based on these observations, exploratory synthesis of PJTE-active inorganic nitrides appears promising for the exploitation of their functional properties. Common d^0^ transition metals warranting investigation and missing from our dataset (some due to our stringent collection/filtering criteria) include Sc^3+^, Ti^4+^, and Zr^4+^; these elements are particularly promising candidates for PJT-induced distortion in inorganic nitrides owing to their low electronegativity.

### Lone-pair stereoactivity

Lone-pair stereoactivity is an electronic phenomenon associated with the observation of highly anisotropic coordination polyhedra for p-block cations with *n*s^2^*n*p^0^ electron configuration. Lone-pair stereoactivity has been described as the causal mechanism for a multitude of material properties not limited to ultra-low thermal conductivity (a property most relevant to the development of next-generation thermoelectrics^168^),^169^ second-harmonic generation response,^170–172^ piezoelectricity,^61,173^ pyroelectricity,^173,174^ ferroelectricity,^175,176^ ferromagnetism,^177^ multiferroicity,^178^ dielectric behavior,^179^ photocatalysis,^157,180^ and the photovoltaic effect.^181^

Lone-pair (LP) stereoactivity originates from strong interaction between the cation s and anion p orbitals leading to a high energy antibonding state which, *via* distortion of the structure, may interact with empty cation p orbitals to form a localized electronic state where the lone pair resides.^182^ As such, LP stereoactivity essentially amounts to a special case of the PJTE (see above), where crucial variables include a (vibronic) distortion mode with net positive overlap between the cation p and mixed cation s and anion p states, and favorable interaction between cation s and p states for the formation of the interacting antibonding state (the energy of which being strongly dependent on that of the anion p states). Thus, LP stereoactivity (*vs.* inertness) is strongly a function of ligand identity; for example, the increasing energy of p states with increasing period has been demonstrated to reduce mixing with cation s states to the point of quenching the effect.^183,184^ It follows that anions have a “sweet spot” of cation s and p atomic orbital energies for which the occurrence and magnitude of lone-pair stereoactivity is maximized, where the initial cation s and anion p interaction determines how much stabilization (if any) may be achieved *via* interaction with empty cation p orbitals. Other factors influencing the occurrence and magnitude of lone-pair stereoactivity include the s character of the antibonding orbital (the higher the s character of the antibonding state, the better it is stabilized *via* mixing with the cation p state) and relativistic effects for period 6 cations, whereby relativistic contraction of the 6s orbital reduces mixing with anion p states, leading to more diffuse lone pairs and less distorted structures.^185^

Much like the PJTE, predicting the occurrence of LP stereoactivity for a given composition/structure requires consideration of orbital energy, symmetry and spatial overlap, thus eludes simple crystal-chemical principles; however, trends may be derived to maximize the probability of observing this phenomenon in new syntheses where full composition and/or structure is not known *a priori*. Trends in atomic orbital energy are particularly relevant in this regard. DFT calculations for a series of simple oxides have shown that the energy of the O 2p state cuts in-between that of the s state of group 13 metals for periods 5 and 6 in way that leaves mixing with In^+^ (group 14) unfavorable and Tl^+^ (group 15) marginally favorable.^182,186^ Mixing becomes more favorable with increasing group number along these periods (*i.e.* with decreasing energy of the metal s state), making group 14 and 15 cations (Sn^2+^/Sb^3+^ and Pb^2+^/Bi^3+^, bar relativistic effects) most susceptible to stereoactive lone-pair formation when bonded to O^2−^. By this principle, the relatively higher-energy s state of period 4 p-block elements may be inferred to require further decrease in energy to best mix with the O 2p orbitals, thus making group 15 and 16 most prone to LP stereoactivity (particularly As^3+^) for this period. Such propositions are in-line with the results of the bond-length dispersion analysis of Gagné & Hawthorne for non-metals^70^ and metalloids/post-transition metals.^71^

Considering that the 2p states of N are slightly more energetic than those of O (due to its lower electronegativity), we presume that LP stereoactivity may be possible for cations with higher-energy states such as In^+^/Ge^2+^, is most likely for ions Sn^2+^, As^3+^, Sb^3+^ and Se^4+^, and is less likely for late-period, higher group elements.

There are unfortunately too few data available to verify this statement; while literature abounds with well-characterized structures containing one or more stereoactive lone pairs for oxides and oxysalts, and to a lesser extent chalcogenides and halides, very few structures have been reported with stereoactive lone-pair electrons when bonded to N^3−^. Presumably, this paucity of data is due to the relatively nascent exploration of (functional) inorganic nitrides. Our dataset contains no data for As^3+^, Se^4+^, Sb^3+^, Te^4+^ Bi^3+^, or any relevant group 17 cations. Only one coordination polyhedron was recorded for Sn^2+^, in Li(Sn(NH_2_)_3_) (50467),^187^ with three short bonds 2.128, 2.132 and 2.170 Å in length and a stereoactive lone pair occupying the 4^th^ apex of a tetrahedron. For period 6 ions, eight coordination polyhedra were recorded for Tl^+^ and two for Pb^2+^; in all cases, short bonds are concentrated in one hemisphere, away from the lone pair, while long bonds (if any) are present in the other. Thus, all p-block cations with *n*s^2^*n*p^0^ electron configuration are observed with a stereoactive lone-pair in the present dataset, in sharp contrast to oxides and oxysalts; this result is promising for the exploitation of the functional properties resulting from lone-pair stereoactivity in inorganic nitrides. Lone-pair stereoactivity is further observed in coordination complexes for Ge^2+^,^188^ and in mixed coordination with Cl^−^ for Se^4+^ (ref. 189) and Te^4+^,^190,191^ showcasing additional opportunities for the solid state.

### The ^[4]^M^6+^ configuration

**Fig. 6:**
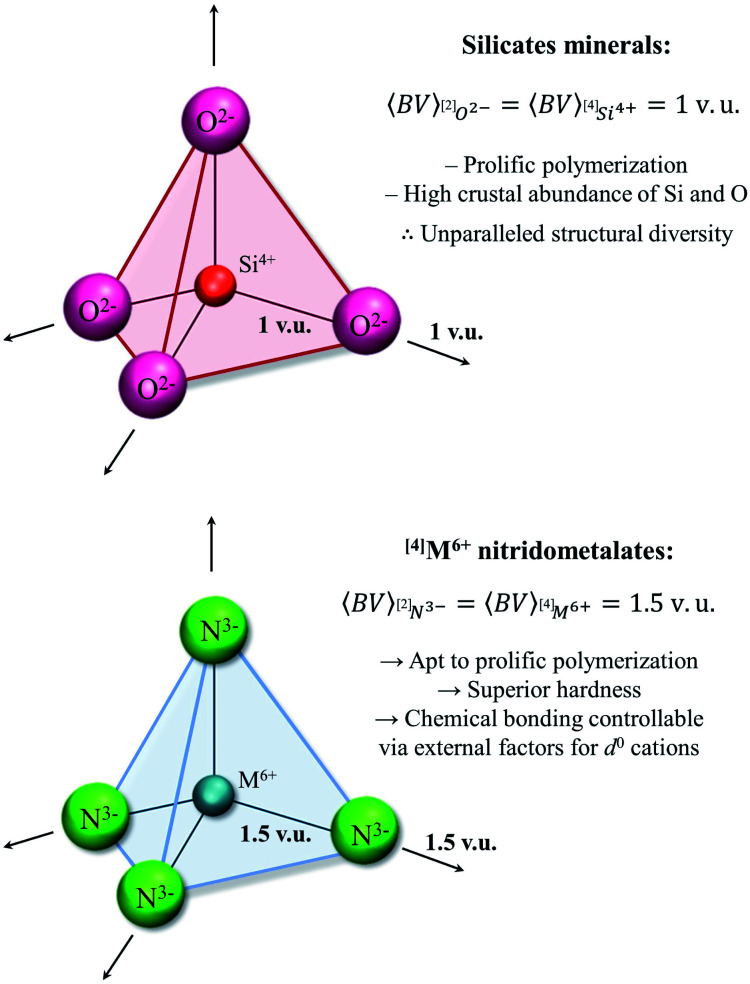
Comparison of the bond-valence constraints between polymerization units [SiO_4_]^4−^ and [M^6+^N_4_]^6−^; in addition to matching the prolific potential for polymerization of silicate minerals, ^[4]^M^6+^ nitridometalates necessarily form harder compounds, and may be tuned *via* external factors.

Earth's remarkable mineral diversity has been attributed to a number of factors spanning geochemical, crystal-chemical and thermodynamic considerations including efficient elemental concentration mechanisms, peculiar electronic behavior for certain elements, mineral stability ranges, *etc.*^83,192,193^ A less-discussed factor enabling mineral diversity is the ability of a (strongly bonded) structural unit to polymerize into a variety of motifs (*e.g.* as oligomers, chains, rings, clusters, sheets, frameworks) inter-linked *via* weakly bonded constituents. This concept is best illustrated for silicate minerals; there are currently 5600+ minerals approved by the International Mineralogical Association, 1550+ of which contain Si and O as essential elements (http://rruff.info/ima/). What are the salient characteristics of the SiO_4_ unit that lead to such remarkable diversity? From the valence-sum rule (eqn (2)), we may deduce that the two bonds made by a bridging ^[2]^O^2−^ ion are ideally 1 v.u. in strength. Similarly, ^[4]^Si^4+^ ideally makes four bonds 1 v.u. in strength. With the bond-valence requirements of ^[4]^Si^4+^ perfectly matching those of ^[2]^O^2−^ (see the *valence-matching principle*, above), the SiO_4_ unit freely polymerizes *via* one, two, three or four corners in a theoretically infinite number of motifs, leading to a remarkably stable and diverse class of minerals that accounts for ∼90% of Earth's crustal composition by volume.^194^ In inorganic nitrides, the analogous bond-valence requirements for a bridging ^[2]^N^3−^ anion are 2 × 1.5 v.u.; thus, hexavalent cations are required for polymerization of corner-sharing tetrahedra to result in proliferation of structural motifs (*i.e.* 4 × 1.5. v.u. = 6 v.u.), hence the significance of the ^[4]^M^6+^ configuration in inorganic nitrides (Fig. 6).

Porous materials (*e.g.* zeolites) are an important class of functional materials for which corner-sharing polymerization of the M^6+^N_4_ unit shows promise for exploratory synthesis. Zeolites are known for their remarkable catalytic and sorption characteristics,^195,196^ offering the ability to carry and optimize specific reactions *via* partial substitution of the cations making up the structural unit according to their Lewis acidity (Table 1).^197^ Although framework structures based on the M^6+^N_4_ unit have been synthesized,^198^ successful incorporation of N^3−^ as the major anion in a zeolite structure has so far only been achieved in the presence of stoichiometric oxygen for oxynitridophosphates (in NPO^199^ and NPT^200^), and otherwise for zeolite-like (pore-less) nitridophosphates^201^ and nitridosilicates.^202^ Interestingly, framework structures based on the M^6+^N_4_ units may exhibit further interesting properties as a result of the pseudo Jahn–Teller effect for d^0^ transition metals such as Mo^6+^ and W^6+^ (*e.g.* ferroelectricity, ferromagnetism, piezoelectricity, magnetic-dielectric bistability; see above), thus possibly providing external control on molecular selectivity for catalytic reactions and adsorption/desorption processes.

Polymerization of the M^6+^N_4_ unit also shows promise for the synthesis of ultrahard materials. The hardness of inorganic nitrides is known to be intimately linked to their bond strength;^203,204^ for example, the hardness of cubic BN (boron nitride), where all bonds are 0.75 v.u. in strength, is only slightly lesser than that of diamond (with bulk modulus 400 *vs.* 440 GPA, respectively).^205,206^ Notwithstanding synthetic feats, the synthesis of M^6+^N_2_ nitrides with bonds 1.5. v.u. in strength (*e.g.* Mo^6+^N_2_ and W^6+^N_2_, analogous to SiO_2_) could lead to new ultrahard materials and ultra-wide-band-gap semiconductors. In addition to their refractory nature, the characteristically high hardness of inorganic nitrides has already been applied to the development of reinforced cements and concretes for different forms of BN.^207,208^

We observe five ions with ^[4]^M^6+^ configuration in our dataset: S^6+^, Cr^6+^, Se^6+^, Mo^6+^ and W^6+^. Both the ^[4]^S^6+^ and ^[4]^Se^6+^ configurations are only observed in oxynitrides thus far, either as [M^6+^O_2_N_2_]^4−^ or [M^6+^O_3_N]^3−^, while the [Cr^6+^N_4_]^6−^ unit has yet to be observed as a product of polymerization. More interesting is the [WN_4_]^6−^ unit, which is observed to polymerize into dimers in LiBa_4_[W_2_^6+^N_7_] (74823),^167^ 6-membered rings in K_14_W_6_^6+^N_16_NH (75033),^209^ chains in Na_5_X[(W^6+^N_3_)_2_] with X = Rb^+^ or Cs^+^ (55534),^210^ sheets in Na_2_K_13_[W_7_^6+^N_19_] (81764)^211^ and into a framework structure in Cs_5_[Na(W_4_^6+^N_10_)] (50002).^212^ Fewer works have studied the polymerization of the [MoN_4_]^6−^ unit; dimers have been described in LiBa_4_[Mo_2_^6+^N_7_]·BaCl_2_ (72400)^213^ and in LiBa_4_[Mo_2_^6+^N_7_] (74822).^167^ Evidently, much of the potential of the ^[4]^M^6+^ configuration in inorganic nitrides lies ahead.

## Conclusion

Following several decades of progressive syntheses primarily driven by new and improved methods of preparation, inorganic nitrides recently matured into a thriving class of inorganic solids with a promising set of functional properties akin to oxide and oxysalt compounds. This work uses structure–property relationships to identify some of the most promising uncharted compositional spaces for inorganic nitrides bearing functional properties, and further provides basic, universal parameters helpful to the verification of high-throughput computational results and the design and characterization of nitrides *sensu lato*.

Whereby chemical-bonding insight gained in the bulk carries well into the study of the local scale, an immediate opportunity enabled by the crystal-chemical data and analyses provided herein regards the study of point defects in semiconductors, more precisely, the (bottleneck) activation of their useful properties *via* extrinsic doping. Considering that doping success typically relies on comfortable substitutional incorporation of foreign ions into the host crystal structure, a logical next step may be to use the data and insight provided herein as stepping stones toward an atomistic understanding of the factors underlying ion substitution in solids – a problem whose solution would fast-track the development of next-generation solar cells, battery materials, electronics, and the many more semiconductor-based technologies we've come to depend on.

Further developments for this class of compounds will surely follow from strong symbiosis between theoretical, synthetic and computational chemists by way of identifying, understanding, and exploiting the structural underpinnings of their functional properties and energetics.

## Notes

Relevant ICSD codes included in parentheses in text.

## Funding sources

This work was supported by a Banting post-doctoral fellowship from the Natural Sciences and Engineering Research Council of Canada, and a Carnegie post-doctoral fellowship from the Carnegie Institution for Science to OCG.

## Conflicts of interest

There are no conflicts to declare.

## Supplementary Material

SC-012-D0SC06028C-s001
