# Supplementary material for: On the crystal chemistry of inorganic nitrides: crystal-chemical parameters, bonding behavior, and opportunities in the exploration of their compositional space
Source: Chem Sci. 2021 Feb 15;12(12):4599–622. doi: 10.1039/d0sc06028c (PMC8179496; doi:10.1039/d0sc06028c)
Supplement: SC-012-D0SC06028C-s001 [file SC-012-D0SC06028C-s001.pdf]

Electronic Supplementary Material (ESI) for Chemical Science.
This journal is © The Royal Society of Chemistry 2021

SUPPORTING INFORMATION

Methods

Mixed-anion coordination polyhedra

Although bond-valence parameters have historically been derived using cation coordination polyhedra with only one kind of anion (e.g. BN_3), it is possible to derive bond-valence parameters using mixed-anion coordination polyhedra, provided that (1) reliable bond-valence parameters are available for the cation-anion pair(s) that does not enter the refinement, and (2) each crystallographically-distinct anion site is fully occupied by a single anion. For example, one may use bond lengths from a BON_2 coordination polyhedron[1] to refine B-N bond-valence parameters, treating the B-O bond-valence as a constant calculated using the parameters of Gagné & Hawthorne.[2] Due to a general paucity of data for nitride compounds relative to oxides and oxysalts, data for coordination polyhedra containing both O^{2-} and N^{3-} were also collected. Below, we show for the first time that the use of mixed-anion coordination polyhedra leads to the same bond-valence parameters as those derived with one type of anion, thus allowing the derivation of bond-valence parameters for cations with otherwise no data.

Bond-valence parameters for mixed-anion polyhedra

Bond-valence parameters were refined with the GRG method for Ba^{2+}, Cr^{3+} and Cu^{2+} with and without their mixed-anion (N^{3-} and O^{2-}) coordination polyhedra to test whether use of mixed-anion coordination polyhedra leads to the same bond-valence parameters as those derived with only one type of anion. For Ba^{2+}, R_o = 2.432 Å and B = 0.405 Å, n = 53, including 9 coordination polyhedra with one or more oxygen anion. Removing those 9 coordination polyhedra, R_o and B refine to 2.433 and 0.406 Å, respectively. For Cr^{3+}, R_o = 1.796 Å and B = 0.403 Å, n = 26, including 14 coordination polyhedra with one or more oxygen anion. Their removal from the optimization gives R_o = 1.796 Å and B = 0.406 Å. For Cu^{2+}, R_o = 1.577 Å and B = 0.515 Å, n = 17, including 13 coordination polyhedra with one or more oxygen anion. Their removal gives R_o = 1.585 Å and B = 0.490 Å. These results show that using mixed-anion coordination polyhedra for refining new bond-valence parameters is justified, provided that the bond-valence parameters used to convert the concerned bond lengths into constants that do not enter the refinement are known to be of high quality. Thus, mixed-anion coordination polyhedra were used in deriving the new set of cation-N^{3-} bond-valence parameters below (Table 1).

Bond-valence parameters for cations observed in multiple coordination numbers

To refine both bond-valence parameters R_o and B for an ion pair (see equation 1), such ion pair must be observed in at least two different coordination environments, either in terms of cation coordination number, or varying mixed-anion ratio. Where this is not observed, either R_o or B must be fixed, and the other parameter refined. In our dataset, 45 cations are observed in more than one coordination number. Of those, 37 have enough data (and data of high enough quality) for confident derivation of their bond-valence parameters, and an additional 3 (Gd^{3+}, P^{5+} and S^{6+}) have varying mixed-anion ratios that allow refinement of both R_o and B despite occurring in only one coordination number. Together, they account for 1168 of the 1436 coordination polyhedra in our final dataset. Bond-valence parameters for these 40 cations were derived optimizing a 2:1 ratio between equations (3) and (4), as described above, and are given in Table 1.

We then plotted the ratio of bond-valence parameter R_o and the observed mean bond-length for the cations $\langle R_{ij} \rangle_{CN}$ (weighting each coordination number equally, using only coordination polyhedra where the cation is bonded solely to N^{3-}) to the nth ionization energy of the cation, a relation first identified by Gagné & Hawthorne.[2] This is shown in Fig. S1, with best-fit equation

$$\frac{R_o}{\langle R_{ij} \rangle_{CN}} = 7.28\ x\ 10^{-3}\sqrt{IE} + 0.526 \quad . \qquad \text{(eq. S1)}$$

Excellent agreement is observed with R^2 = 0.86, which increases to R^2 = 0.92 when removing data points for Ag^+ and Li^+ (Gagné & Hawthorne found R^2 = 0.75 for O^{2-}, for 90 cations[2]). Thus, the validity of using this relation to extrapolate values of R_o for cations observed in only one coordination number is confirmed.

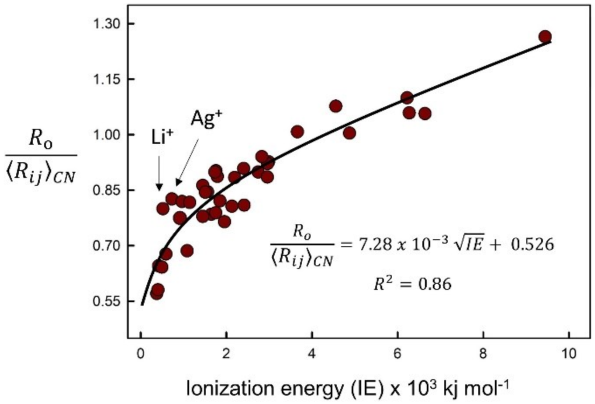

Figure S1: Relation between the ratio of bond-valence parameter R_o and the coordination-based mean bond-length for the cation as a function of ionization energy (kj mol^{-1}).

In some cases, deviation between the value of R_o predicted by eq.5 and that obtained from the GRG refinement results from the shallowness of the global minimum of the RMSD, and is an artifact of small sample size. For example, although Cs^+ (n = 24) has the largest deviation between refined (1.979 Å) and predicted (2.315 Å) R_o, the associated RMSD values are 0.074 v.u and 0.098 v.u., respectively, compared to an ideal bond-valence sum of 1 v.u. for Cs^+. Most values of R_o in the range quoted above could be suitable provided that the B parameter is properly refined.

Bond-valence parameters for cations observed in one coordination number

Bond-valence parameters for the 36 ion pairs observed in only one coordination number, or for which not enough data are available to refine both bond-valence parameters with confidence, were derived in the following way:

(1) Fix R_o to the value predicted by equation (S1). Let B refine via the GRG method. If the value for B falls within a range similar to that of ions with similar crystal-chemical behavior, accept the bond-valence parameters. Otherwise, move on to (2);

(2) Fix B to the family average (e.g. 0.399 Å for the transition metals) or to a value compatible with ions of similar crystal-chemical behavior. Let R_o refine and see if it falls within a reasonable range (typically within a 6.0% difference, the average deviation between observed and predicted values of R_o for the 40 cations for which both R_o and B were refined). If not, move on to (3);

(3) Fix B to its mean value for all families combined (0.422 Å) and let R_o refine. This is typically done where there are insufficient data available to make a reasonable estimate of B (e.g. for actinides U^{4+} and U^{6+}).

As discussed in Gagné & Hawthorne,[2] fixing the value of R_o is less forgiving than fixing that of B and should be done with caution. For the 36 cations considered here, we fixed R_o for 4 and B for 32 ion pairs (the method of derivation is identified in Table 1).

Anion sum verification

For simplicity, bond-valence parameters are usually derived by optimizing the valence-sum rule for cations (the work of Krivovichev & Brown[3] being a notable exception). However, we emphasize that the valence-sum rule applies equally to cations and anions, and that good agreement for cation bond-valence sums in no way implies good agreement for anion bond-valence sums (and vice versa). It is imperative that new bond-valence parameters be checked against *both* these quantities, on a large set of crystal structures, before they should be widely accepted. Of the 70+ publications that have given bond-valence parameters since the inception of the model (https://www.iucr.org/__data/assets/file/0007/126574/ bvparm2016.cif), such verification has only been done by Krivovichev & Brown for the Pb^{2+}-O^{2-} ion pair,[3] by Gagné & Hawthorne for their comprehensive set of bond-valence parameters for cations bonded to O^{2-},[2] and by Sidey & Shteyfan for the P^{5+}-S^{2-} ion pair.[4]

We assembled a set of structures with the goal of evaluating as many bond-valence parameters as possible from Table 1 (see Table S1), i.e., unless no structure could be evaluated using solely the bond-valence parameters derived in this work. The RMSD for the anion bond-valence sums (BVS) over the resulting set of 52 crystal structures, covering 135 anion coordination polyhedra, is 0.209 v.u. The simple mean deviation is 0.172 v.u., or 5.7% compared to an ideal BVS of 3 v.u. For comparison, the mean *cation* RMSD for ion pairs with 10 or more coordination polyhedra is 0.122 v.u. (0.120 v.u. over all ion pairs weighted by their number of coordination polyhedra). This is very similar to the value reported by Gagné & Hawthorne for O^{2-},[2] of 0.126 v.u. for $n \geq 10$, (0.124 for all data weighted by their number of coordination polyhedra). These authors also reported a RMSD of 0.104 v.u. over 511 anion coordination polyhedra of O^{2-} (5.2% compared to an ideal BVS of 2 v.u.).

These numbers allow us to make two observations. First, the RMSD of the anion BVS is slightly larger than that of cations. This result is somewhat expected; aside from *not* being the subject of the optimization, few structures could be evaluated for anion BVS, and those that could were not necessarily ideally suited to the task (e.g. $0.3 < R_1 < 0.06$). Second, we observe slightly higher RMSD values for both cation and anion BVS for structures of N^{3-} vs O^{2-}. This is likely a consequence of sampling a wide range of meta-stable nitride structures,[5] as opposed to thermodynamically-stable minerals which make up a greater fraction of the oxide and oxysalt data of Gagné & Hawthorne.[2] Meta-stable structures generally entail less-than-perfect mapping of bond-length constraints in three-dimensional space (which can be calculated *a priori*[6]) under the constraints of symmetry and periodicity, leading to higher variations in mean bond lengths across structure types in comparison to thermodynamically-stable structures.[7]

References

1. Schmid, S. & Schnick, W. Synthese und kristallstruktur des ersten oxonitridoborates — $Sr_3[B_3O_3N_3]$. *Z. Für Anorg. Allg. Chem.* **628**, 1192 (2002).
2. Gagné, O. C. & Hawthorne, F. C. Comprehensive derivation of bond-valence parameters for ion pairs involving oxygen. *Acta Crystallogr. Sect. B Struct. Sci. Cryst. Eng. Mater.* **71**, 562–578 (2015).
3. Krivovichev, S. V. & Brown, I. D. Are the compressive effects of encapsulation an artifact of the bond valence parameters? *Z. Für Krist. - Cryst. Mater.* **216**, (2001).
4. Sidey, V. & Shteyfan, A. Revised bond valence parameters for the P^{+5}/S^{-2} ion pair. *J. Phys. Chem. Solids* **103**, 73–75 (2017).
5. Sun, W. *et al.* Thermodynamic routes to novel metastable nitrogen-rich nitrides. *Chem. Mater.* **29**, 6936–6946 (2017).
6. Gagné, O. C., Mercier, P. H. J. & Hawthorne, F. C. *A priori* bond-valence and bond-length calculations in rock-forming minerals. *Acta Crystallogr. Sect. B Struct. Sci. Cryst. Eng. Mater.* **74**, 470–482 (2018).
7. Gagné, O. C. & Hawthorne, F. C. Mean bond-length variations in crystals for ions bonded to oxygen. *Acta Crystallogr. Sect. B Struct. Sci. Cryst. Eng. Mater.* **73**, 1019–1031 (2017).

Data

Table S1: ICSD code for the structures used in the anion bond-valence sum verification

Cation	ICSD code
H^{+}	63027
Li^{+}	67560
Be^{2+}	402341
B^{3+}	41022
C^{4+}	59860
N^{5+}	402743
Na^{+}	401210
Mg^{2+}	411175
Al^{3+}	34475
Si^{4+}	34096
P^{5+}	415715
S^{4+} and S^{6+}	14151
K^{+}	81528
Ca^{2+}	107304
V^{3+}	41060
V^{5+}	409501
Cr^{3+}	40205
Cr^{5+}	82360
Cr^{6+}	66097
Mn^{2+}	281280
Mn^{3+}	67888
Mn^{5+}	280693
Fe^{2+}	72389
Fe^{3+}	68523
Co^{+}	72387
Rb^{+}	81529
Cu^{+}	86066
Zn^{2+}	80376
Ga^{3+}	86016
Se^{6+}	108857
Sr^{2+}	71059
Nb^{5+}	412060
Mo^{6+}	409473
Ag^{+}	23111
Cd^{2+}	4097
Cs^{+}	72546
Ba^{2+}	71060
La^{3+}	411742
Ce^{3+}	402910
Ce^{4+}	74791

Pr^{3+}	72107
Nd^{3+}	410848
Sm^{3+}	240312
Eu^{2+}	59257
Er^{3+}	84706
Yb^{3+}	59258
Lu^{3+}	240311
Hf^{4+}	97997
Ta^{5+}	412585
W^{6+}	409472
Pb^{2+}	410915

Table S2 : *A priori* bond valences for crystal structures used in text

$K_2CuFe(CN)_6$	*K*	*Cu*	*Fe, C*1-*C*3	∑
*N*1	0.089 ×4↓×4→	0.321 ×2↓	2.321 ×2↓	3
*N*2	0.107 ×3↓×3→	0.339 ×2↓	2.339 ×2↓	3
*N*3	0.107 ×3↓×3→	0.339 ×2↓	2.339 ×2↓	3
∑	1	2	14	

Hf_3N_4	*Hf*	∑
N	0.5 ×8↓ ×6→	3
∑	4	

$Li_4(TaN_3)$	*Li*1	*Li*2	*Li*3	*Ta*	∑
*N*1	0.167 ×2↓ ×4→			1.167 ×2↓ ×2→	3
*N*2	0.333 ×2↓ ×2→	0.25 ×4↓ ×2→	0.25 ×4↓ ×2→	1.333 ×2↓	3
∑	1	1	1	5	

$LiBa_4(M^{6+}{}_2N_7)$<br>M^{6+} = W, Mo	*Li*	*Ba*1	*Ba*2	*Ba*3	*Ba*4	Ba5	*M*1	*M*2	∑
*N*1		0.268 ×2↓ ×2→	0.316	0.315	0.299 ×2↓			1.535	3
*N*2		0.268	0.317	0.315 ×2↓ ×2→		0.212 ×2↓	1.573		3
*N*3				0.099 ×2→	0.083 ×2↓ ×2→			1.318 ×2→	3
*N*4		0.331 ×2↓ ×2→	0.379		0.362 ×2↓			1.597	3
*N*5	0.249 ×2↓ ×2→	0.252	0.301			0.196 ×4↓ ×2→	1.557		3
*N*6	0.280	0.283	0.331	0.330		0.227 ×2↓		1.550	3
*N*7			0.082 ×2→	0.080 ×2→			1.338 ×2→		3
*N*8	0.223		0.274	0.273 ×2↓ ×2→	0.257 ×2↓	0.170 ×2↓	1.531		3
∑	1	2	2	2	2	2	6	6	
